# An integrated multi-scale framework for economically feasible landscape connectivity in working agricultural systems

**DOI:** 10.1038/s44264-026-00166-5

**Published:** 2026-10-30

**Authors:** Kevin Bradley Dsouza, Graham Alexander Watt, Yuri Leonenko, Juan Moreno-Cruz

**Affiliations:** 1 Department of Earth and Environmental Sciences, University of Waterloo, Waterloo, ON Canada; 2 Royal Bank of Canada, Toronto, ON Canada; 3 Department of Geography and Environmental Management, University of Waterloo, Waterloo, ON Canada; 4 School of Environment, Enterprise and Development, University of Waterloo, Waterloo, ON Canada

**Keywords:** Ecology, Ecology, Environmental sciences, Environmental social sciences

## Abstract

Agricultural landscapes must simultaneously sustain food production, support rural economies, and maintain ecological resilience. However, decades of landscape simplification have reduced semi-natural habitats, fragmented ecological networks, and weakened the ecosystem services that underpin agricultural productivity. Existing approaches to ecological intensification improve some farm-level services but do not generate the spatial coordination required for landscape-scale ecological function. Here, we present an integrated modeling framework that links farm-level ecological intensification, spatial habitat connectivity optimization, and a Bayesian search over realistic agri-environmental policies. Applied to a real agricultural landscape, the framework identifies feasible pathways for enhancing ecological function while maintaining economic viability. By explicitly connecting the scales at which farmers make decisions, ecosystems operate, and policies are implemented, this approach reveals how coordinated restoration can transform fragmented agricultural mosaics into more resilient, multifunctional landscapes. Our results demonstrate the potential for policy design to enable ecologically strategic and economically feasible habitat restoration, offering a pathway toward more sustainable agricultural systems.

## Introduction

Agricultural landscapes today are at the center of multiple, intensifying pressures. They must reliably produce food for growing populations, remain economically viable for farmers and rural communities, and sustain the ecological processes that underpin long-term productivity. Yet decades of agricultural intensification have simplified these landscapes, reduced the extent and diversity of semi-natural habitats, and fragmented previously continuous ecological networks. Such structural changes have contributed to declines in biodiversity, weakened ecosystem functions, and increased the vulnerability of agricultural systems to environmental stressors^1–3^. Ecosystem services such as pollination, natural pest control, nutrient cycling, and hydrological regulation depend on the spatial distribution and quality of habitat across the landscape. When these habitats become isolated or sparse, the ecological flows that support agricultural production diminish, threatening yield stability and resilience^4–7^. Ensuring sustainability in working agricultural systems, therefore, requires approaches that enhance ecological integrity without imposing untenable economic burdens on producers.

Ecological intensification (EI) offers one promising pathway for reconciling productivity and ecological health. EI seeks to enhance agricultural output by relying more heavily on ecological processes and less on synthetic inputs^1,2,8^. A substantial body of empirical work has demonstrated that services such as pollination and biological control exhibit strong spatial dependence, with benefits declining rapidly as the distance between crops and semi-natural habitat increases^4,5,9–11^. These services can increase yields, reduce production risk, and help stabilize output under environmental variability. However, EI is inherently a farm-level strategy: farmers adopt habitat restoration or biodiversity-friendly practices based on their private assessments of costs and expected gains^12^. The cumulative landscape-level effects of EI therefore emerge from decentralized decisions that do not necessarily align with collective ecological goals^13^. Even where EI is profitable for many farms, the resulting habitat configuration often remains patchy and insufficient to sustain ecological connectivity^9,14–16^. Thus, while EI can deliver important local benefits, it rarely produces the coordinated spatial patterns required for landscape-scale ecological function. In this study, ecological intensification (EI) is operationalized through two spatially explicit interventions: enhancement of field margins and habitat conversion. Margin interventions allocate portions of field edges to semi-natural vegetation while maintaining crop production, whereas habitat conversion represents full restoration of selected cropland patches. These interventions differ in opportunity cost and ecological effect, reflecting empirical evidence that both margin enhancements and habitat restoration can strengthen pollination and pest control services in agricultural systems^4,9,15,16^.

Landscape connectivity represents one of the most important yet under-managed dimensions of agricultural sustainability^17^. Connectivity governs the capacity for organisms to move between habitat patches, maintain genetic diversity, track shifting climate conditions, and support functional ecological networks^9,15,18–20^. High connectivity can improve population stability, facilitate species dispersal, and enhance ecosystem resilience. Conversely, fragmented landscapes reduce movement pathways, increase local extinctions, and diminish the effectiveness of ecological services crucial to agriculture. Strategic habitat placement can substantially strengthen connectivity, often through the addition of a small number of well-positioned patches that act as stepping stones or corridors^10,11,16,21,22^. Such targeted interventions can generate ecological benefits that far exceed those of uniformly distributed restoration efforts. Despite these insights, most agri-environmental policies focus on area-based restoration or uniform requirements that do not incorporate the spatial logic of ecological networks^23–26^. As a result, many restoration programs invest in habitat creation that contributes little to improving connectivity, limiting ecological effectiveness and wasting scarce conservation resources.

Scientific advances in landscape ecology, metapopulation dynamics, and conservation planning have increasingly emphasized the importance of spatial configuration in determining ecological outcomes. Connectivity-driven thresholds, non-linear responses to habitat placement, and the existence of keystone patches mean that landscapes can shift rapidly between low-functioning and highly functional ecological states depending on where restoration occurs^11,18,21^. Pollination ecology has shown that the benefits of semi-natural habitat decay steeply with distance, creating strong spatial gradients that determine where restoration is most effective^4^. Biological control studies similarly reveal that natural enemy dispersal processes shape the spatial footprint of pest suppression^10,11,27^. These ecological dynamics highlight that improving sustainability requires more than simply adding habitat; it requires placing habitat in locations where it most effectively enhances ecological flows and supports network coherence. Translating these principles into practice demands modeling frameworks that combine ecological realism, economic feasibility, and policy implementability.

However, existing modeling approaches typically capture only one or two of these dimensions. Bioeconomic models provide valuable insights into farm-level decision-making, opportunity costs, and behavioral responses to policy incentives^28–32^. Yet they often treat ecological processes as simplified yield modifiers that do not depend on spatial context. Conversely, spatial connectivity models identify optimal locations for habitat to maximize ecological objectives^27,33,34^, but they frequently abstract away from economic constraints or assume full control over land allocation. Policy evaluation frameworks assess how different mechanisms influence adoption, cost-effectiveness, or environmental outcomes^24,33,35–40^, but they rarely determine whether those mechanisms can generate spatial patterns that resemble ecological optima. As a result, the ecological, economic, and policy elements of agricultural sustainability remain analytically disconnected. This fragmentation leads to interventions that are either ecologically sound but economically unrealistic, economically feasible but ecologically weak, or administratively simple but poorly aligned with ecological processes.

This gap is not merely conceptual but practical. Real agricultural landscapes are governed by private decision-making, shaped by heterogeneous opportunity costs, and constrained by diverse economic realities. At the same time, they require coordinated spatial interventions to support ecological function at scales relevant for biodiversity, ecosystem services, and climate resilience. A framework that fails to reconcile these layers cannot provide actionable guidance for policy or landscape planning. While existing optimization approaches have studied farm-level decisions, spatial landscape planning, or policy instruments in isolation^12,17,22,29–32,39,41–43^, none have connected all three. This unmet need: linking ecological processes, farmer behavior, and policy design within a single decision-analytic system for real-world sustainability outcomes, motivates the modeling approach developed in this study.

**Fig. 1:**
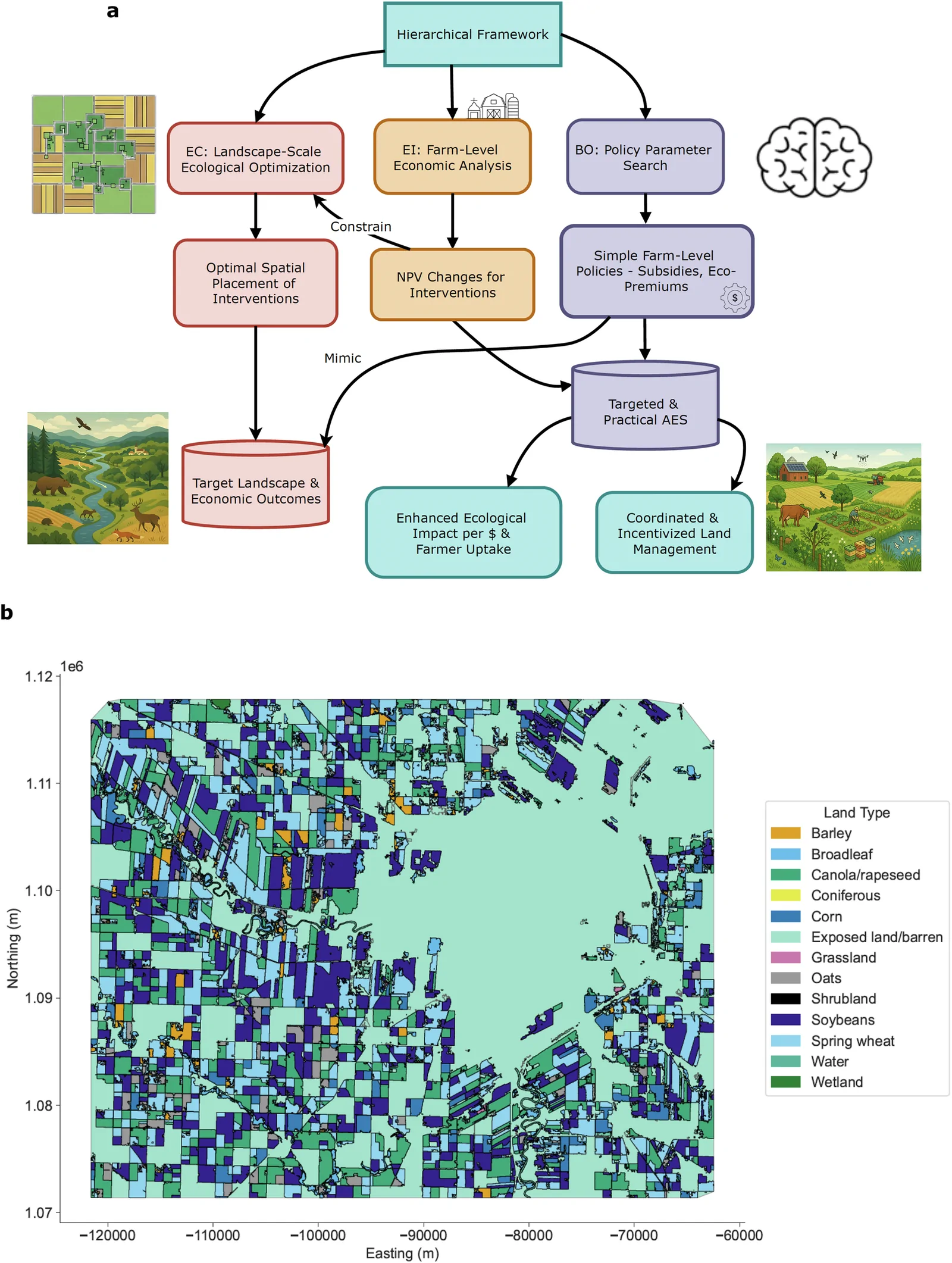
The hierarchical optimization framework and demonstration region. **a** The three-layer hierarchical optimization framework. The framework sequentially links models across scales: (1) a farm-scale Economic Intensification (EI) model determines economically optimal fractions for interventions; (2) a landscape-scale ecological connectivity (EC) model uses these fractions as constraints to optimize the spatial configuration for maximum connectivity; and (3) a Bayesian optimization layer finds simple policy parameters to replicate the spatially optimized outcomes. **b** The agricultural landscape in Manitoba, Canada, was selected as the study area to demonstrate the applicability of the proposed framework.

We propose an integrated system in which economic optima at the farm scale inform spatial optimization at the landscape scale, and in which policy instruments are treated as endogenous decision variables rather than exogenous assumptions. Our hierarchical optimization framework links ecological intensification (EI), ecological connectivity (EC), and Bayesian policy search (BO) into a unified, multi-scale decision architecture. The first component models how individual farms make habitat restoration decisions under ecological intensification, grounded in profit maximization and spatially explicit ecological service functions. This captures the economic reality that farmers will restore habitat only when it does not compromise long-term profitability. The second component identifies where habitat restoration produces the greatest gains in ecological connectivity, using a spatial optimization approach that accounts for graph-theoretic landscape structure and functional ecological flows. This step produces a landscape-scale ecological benchmark that shows what level of connectivity improvement is theoretically attainable under economically acceptable constraints. The third component evaluates which agri-environmental policies: mandatory rules, incentive payments, or hybrid instruments, can induce enough coordinated restoration to approximate this ecological benchmark. These policies operate on behavioral and economic levers, shaping aggregate landscape patterns by influencing thousands of individual decisions. We explicitly link these three components within a single modeling system (Fig. 1a).

In short, our contributions are the following: (a) A Novel Hierarchical Framework: We propose a unified modeling architecture that bridges the gap between farm-level economic decision-making, landscape-scale ecological connectivity, and policy design, enabling the simultaneous optimization of competing objectives across multiple scales; (b) Reconciling Ecology and Economics: We demonstrate that substantial gains in landscape connectivity can be achieved without compromising farm viability by identifying “hubs" that offer high ecological leverage at low economic cost; (c) Goal-Dependent Policy Optimization: We show that there is no single universal policy solution; rather, the optimal mix of mandatory rules and incentives depends on specific societal goals, allowing policymakers to explicitly trade off between cost, connectivity, and producer profitability; and (d) Actionable Pathways for Sustainability: We provide a flexible, adaptable tool for designing context-specific interventions, applicable to diverse governance structures ranging from farmer cooperatives to regional planning authorities.

## Results

### Ecological intensification enhances local services but lacks landscape coordination

The ecological intensification (EI) model produced spatially heterogeneous but economically meaningful restoration outcomes across the study region. Farms differed substantially in the amount of habitat they restored under profit-maximizing behavior, and these differences aligned closely with ecological gradients known to shape service provision in agricultural landscapes. Analysis of plot characteristics revealed weak correlations with optimal intervention strategies (Supplementary Figs. 1 and 2). Farms located near semi-natural habitats or within more structurally complex areas exhibited the strongest economic gains because the yield-enhancing effects of pollination and natural enemy activity were concentrated in these locations.

**Fig. 2:**
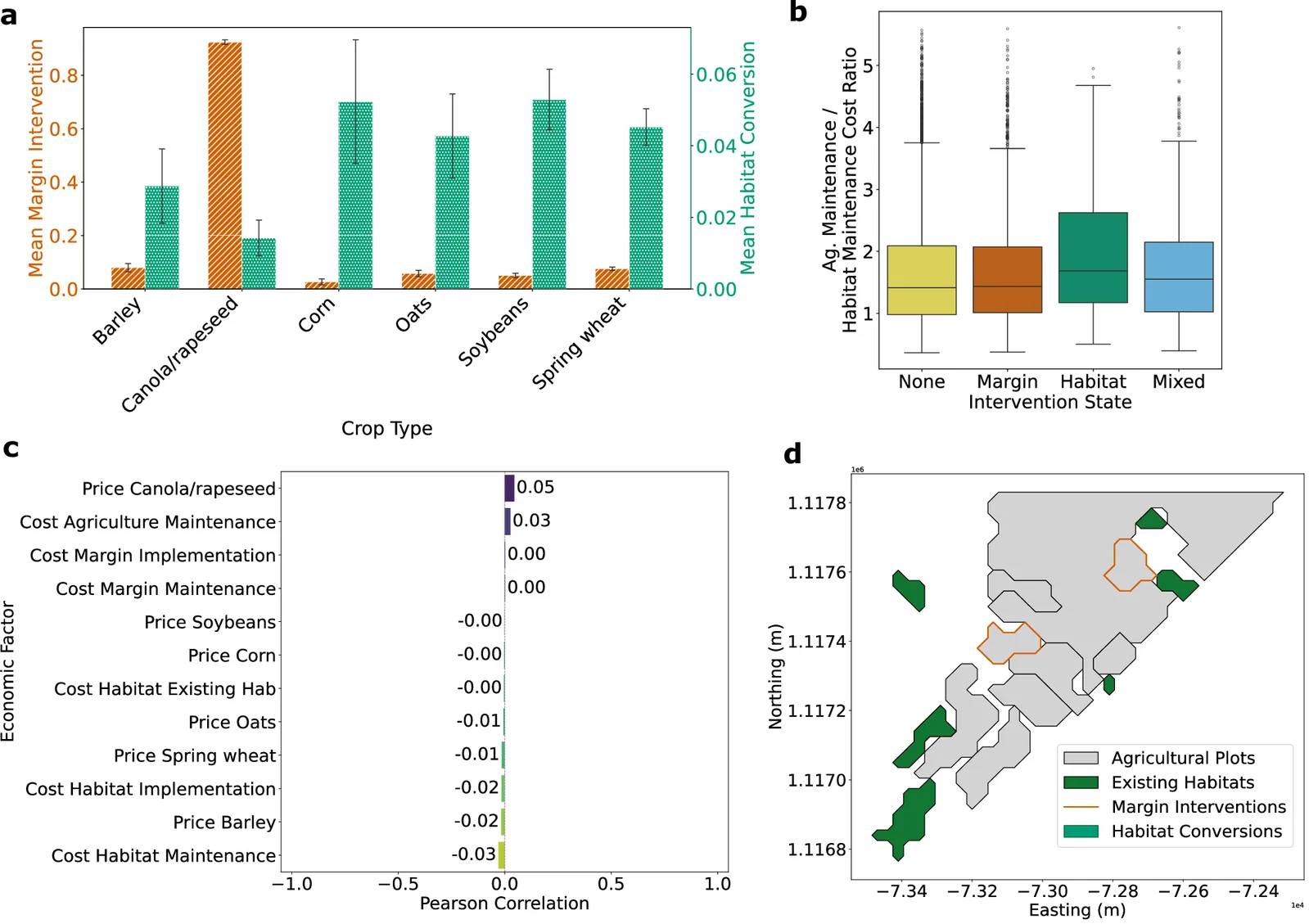
Results from the Economic Intensification (EI) model. **a** Optimal fractions of margin and habitat interventions vary by crop type. Margin interventions are heavily favored in high-value Canola/rapeseed plots, while habitat conversion is prominent, at lower levels, in Soybean and Corn. **b** A box plot of intervention states as a function of the ratio of agricultural maintenance and habitat maintenance costs. Habitat conversion is linked to a high ratio of agricultural maintenance cost relative to habitat maintenance cost. **c** Sensitivity analysis illustrating how economic factors, including crop prices and maintenance costs, influence the level of habitat conversion. **d** An example of an optimal intervention layout determined by the EI model for a selected farm configuration, highlighting the general preference for margin interventions over habitat conversions. In the configuration shown, no habitat conversions occur, and therefore, that shade is absent from the map.

We found that optimal intervention strategies are crop-dependent and differ between intervention types (Fig. 2a). Canola/rapeseed plots distinctly favored margin interventions, showing the highest mean allocation by a large margin. Habitat conversion, while generally implemented at lower levels than margins, was most prominent in Soybean (mean ≈ 0.055) and Corn (mean ≈ 0.05) plots (Fig. 2a). Other crops like Barley, Oats, and Spring Wheat received minimal allocations of either intervention type. This divergence reflects the interplay of parameterized benefits, costs, and crop prices. Higher adoption of interventions in Canola and Soybeans is driven by substantial associated ecosystem service benefits (*α*, *δ*, etc.) and/or high market value (*pc*), which justify the intervention costs.

Economic factors, particularly maintenance costs, significantly influenced the overall intervention strategy chosen. Simulations resulting in habitat conversion (‘Habitat’ states) were associated with higher ratios of agricultural maintenance cost relative to habitat maintenance cost (Fig. 2b and Supplementary Fig. 3). Correspondingly, the decision to implement margins (‘Margin’ state) was linked to lower ratios of margin maintenance cost relative to habitat maintenance cost. Correlation analysis weakly confirmed these trends, showing habitat conversion levels were positively driven by higher agricultural maintenance costs (r=+0.03) and negatively by higher habitat maintenance costs (r= − 0.03) (Fig. 2c and Supplementary Fig. 3). Among other factors, Canola/rapeseed price affected both interventions the highest (r=+0.05), because of its strong ecosystem service benefits (Fig. 2c; Supplementary Figs. 8 and 9).

Despite these local ecological and economic gains, the EI-derived landscape remained insufficient to generate meaningful connectivity at the regional scale. Habitat patches tended to emerge in isolated clusters or short linear segments, increasing ecological function around individual fields but leaving large gaps between major habitat nodes (Fig. 2d). This outcome underscores a core challenge of sustainable landscape design: privately optimal decisions often generate ecologically beneficial changes locally but are rarely sufficient to transform the structural connectivity of the broader landscape.

### Strategic interventions increase connectivity without compromising farm viability

**Fig. 3:**
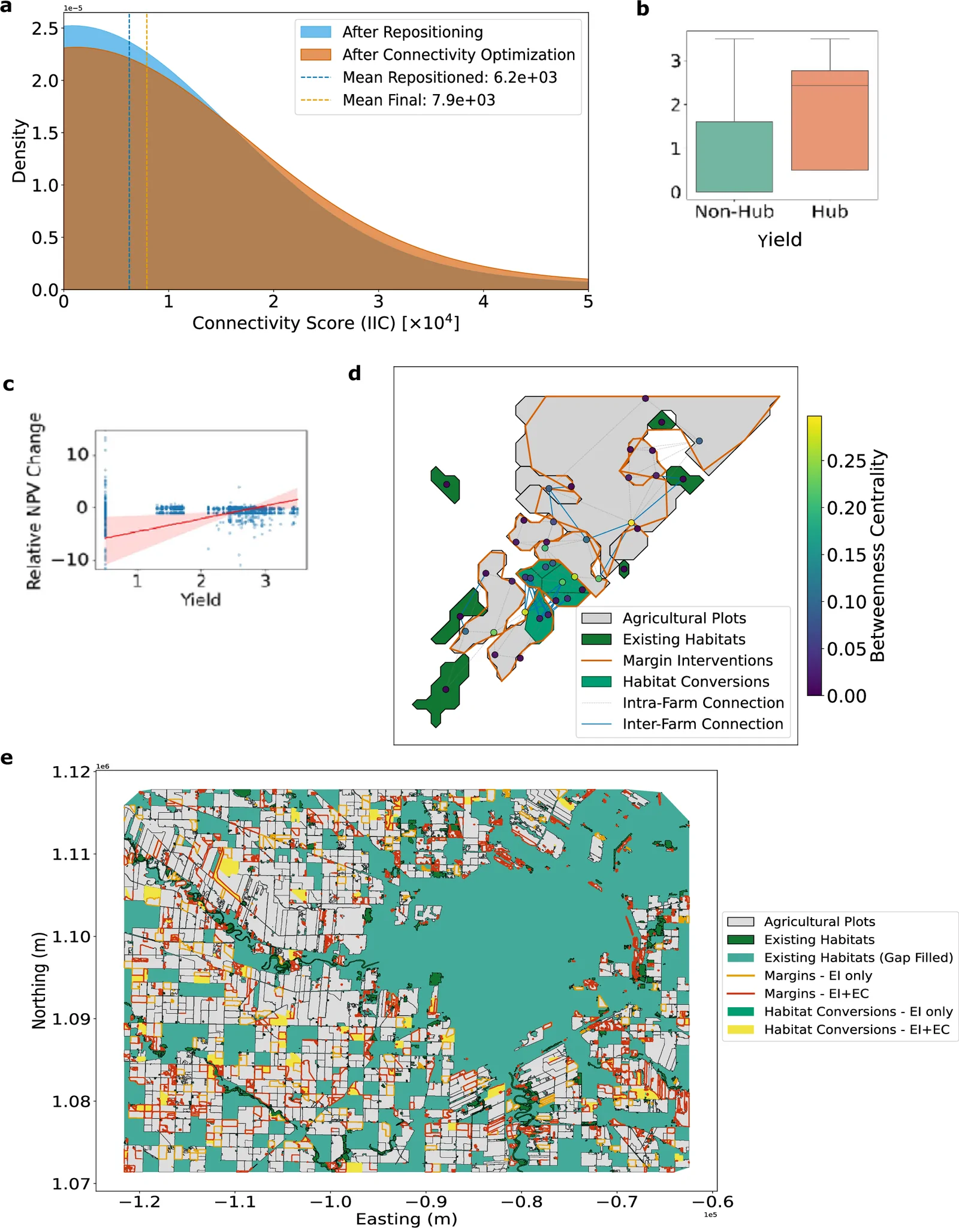
Results of the ecological connectivity (EC) optimization model. **a** The distribution of landscape connectivity scores (IIC) obtained from the configurations after each optimization stage. The full “Connectivity Optimization" stage (red) results in a substantial improvement in landscape connectivity compared to the simpler “Repositioning" of interventions (blue). **b** Box plot of aggregate yield for hub and non-hub plots. Hub plots exhibit a higher median yield, indicating a greater economic capacity to sustain ecological interventions. **c** Scatter plot of relative change in NPV after connectivity optimization compared to EI baseline versus baseline yield. Plots with higher intrinsic yields tend to experience smaller relative NPV reductions. **d** Optimal interventions decided by the EC model for a selected configuration of farms, along with plot-level betweenness centrality and intra-farm (dashed gray)/inter-farm (solid blue) connections. Plot-level betweenness centrality highlights the importance of certain plots as stepping stones in the network. **e** Composite map distinguishing interventions selected during the initial ecological intensification (EI) phase from additional interventions identified during the Connectivity Optimization (EI+EC) phase. The optimization builds upon the locally profitable EI baseline by strategically filling gaps to establish landscape-scale corridors.

The connectivity optimization produced a markedly different landscape configuration. When the optimization was permitted to allocate additional habitat subject to the constraint that no farm’s profitability fell below an economically acceptable threshold, the resulting pattern exhibited far greater ecological coherence. Compared to repositioning purely existing interventions from step 1, connectivity optimization yielded substantially higher IIC scores, with the mean IIC score increasing from 6.3 × 10^3^ after repositioning to 7.9 × 10^3^ after optimization (Fig. 3a). The optimization frequently identified narrow corridors and bridging patches in areas where large habitat clusters were separated by modest distances. These bridging patches, often less than a hectare in size, played an outsized role in shaping network structure. Restoring them lowered the effective distance between clusters to within the dispersal thresholds of key pollinator and natural enemy groups, thereby enabling multi-step ecological movement across what had previously been fragmented regions. While repositioning can offer some improvement by optimizing spatial arrangement, allowing the optimization framework to select both the location and the amount (subject to economic viability) of interventions provides far greater potential for enhancing landscape-scale ecological connectivity (Fig. 3a).

To explore the connectivity-optimized structure further, we analyzed the resulting configuration of habitat patches and field margins. We identified “connectivity hubs” as agricultural plots ranking within the top 10% by aggregated betweenness centrality (Supplementary Fig. 10). Betweenness centrality was calculated on the spatial graph of interventions, defined as the fraction of shortest paths between all node pairs that pass through a given node, and then summed for all intervention nodes within each agricultural plot. This metric quantifies the extent to which a spatial unit lies along ecological pathways connecting habitat patches, identifying locations that serve as critical stepping stones in ecological networks. Such stepping-stone locations play a disproportionate role in maintaining connectivity in fragmented landscapes^27,34,44^. The aggregate analysis revealed notable trends: hub plots tend to have higher median yields (Fig. 3b), more complex shapes (higher perimeter, higher area, more sides), and more neighbors. Hub plots have higher median base yields, potentially because plots with initially higher base yields are better able to economically sustain the implementation of connectivity-enhancing interventions. The yield boost from ecosystem services on these high-yield plots helps to ensure that the farm’s overall profitability (NPV) remains within acceptable limits while the interventions contribute to the landscape connectivity goals. Moreover, Baseline yield exhibited a positive correlation with the relative NPV change (Fig. 3c), suggesting that plots with higher intrinsic yields tend to suffer smaller relative NPV losses. This could be attributed to the optimization process, which, while maximizing landscape-level connectivity, implicitly favors interventions in areas where the opportunity cost (foregone yield from the highest-productivity land) is lower, thereby relatively shielding the highest-yielding plots from negative impacts.

We visualized the resulting network structure (Fig. 3d), showing that high centrality nodes are positioned to bridge gaps and link habitat clusters. Additionally, by distinguishing between locally optimal (EI) and spatially optimized (EI+EC) interventions (Fig. 3e), we observe that the connectivity optimization strategically extends the EI baseline. It adds critical bridging patches to connect the fragmented clusters formed by local profit maximization, creating continuous corridors and stepping stones. Crucially, all connectivity improvements were achieved without violating the economic feasibility constraints. No farm in the optimized configuration lost more than the allowable margin of net present value relative to its EI baseline. This demonstrates that transformative ecological outcomes need not require large-scale cropland retirement or substantial economic sacrifice. Indeed, the spatial leverage inherent in connectivity means that restoring a small, strategically chosen proportion of the landscape can yield benefits that far exceed the cost of the restored area.

### Optimal policy selection depends on specific goals and trade-offs

**Fig. 4:**
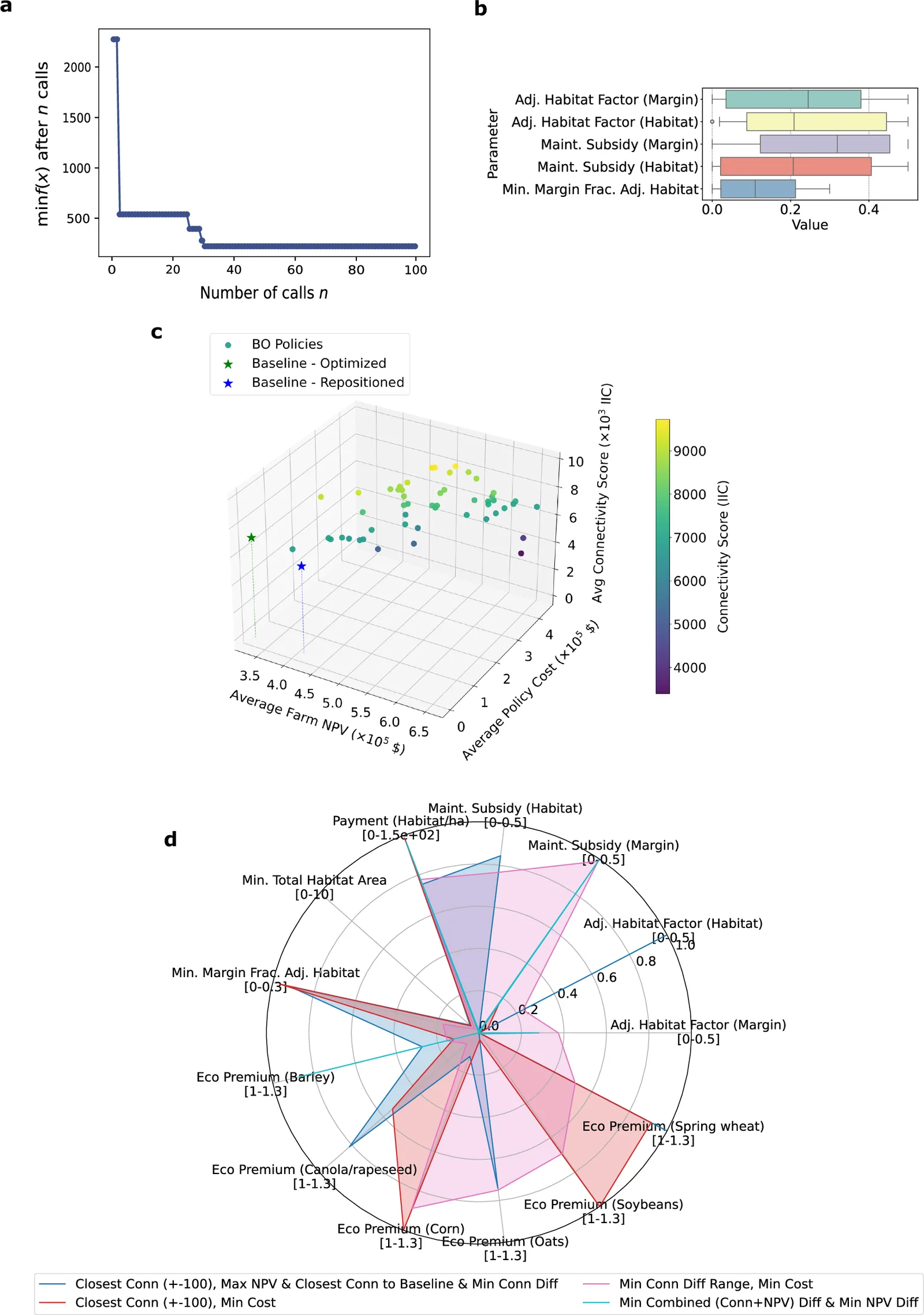
Bayesian optimization (BO) results identify a portfolio of high-performing agri-environmental policies. This figure provides a summary of the results from the BO process, from search dynamics to the characteristics of effective policies. **a** Minimum objective function value found with increasing number of evaluations. The search process is efficient, which rapidly identifies near-optimal solutions within approximately 50 evaluations. **b** Policy parameter distributions in top-performing policies. There is a preference for moderate subsidy levels and relatively low mandatory habitat adjacency requirements, suggesting gentle nudges can be highly effective. **c** Three-dimensional scatter plot visualizing the performance of policies across Average Connectivity (IIC), Average Farm NPV, and Average Policy Cost. The color gradient represents the connectivity score. Optimizing across these three dimensions reveals policies that achieve high connectivity without compromising economic viability. **d** Radar profiles of specific successful policy archetypes, including closest connectivity to baseline, minimum cost and maximum farm NPV. etc. Different strategies, from incentive-heavy approaches using eco-premiums to more frugal, cost-conscious designs, can achieve desirable outcomes.

We employed Bayesian optimization^45^ to find the parameters (*θ*_*P*_) of non-spatially explicit, farm-level EI policy instruments. The convergence plot (Fig. 4a) illustrates the progress of the optimization. It shows the minimum objective function value (average absolute connectivity difference) found as the number of evaluations increased. Significant improvements occurred within the first ~50 calls, after which the rate of improvement slowed, indicating that the BO effectively explored the parameter space and likely converged towards a near-optimal solution within the defined search space and evaluation limit.

Many BO-derived policies achieve average connectivity scores comparable to, or even exceeding, the “Baseline-Optimized” scenario (Green Star). Crucially, these ecological gains are often accompanied by higher average farm Net Present Values (NPVs) compared to this same optimized baseline (Fig. 4c and Supplementary Fig. 11). This improvement in average farm profitability under BO policies is largely because the policy evaluations did not impose the same farm-level NPV constraints that might have limited the “Optimized Baseline”. Therefore, all the policies on the top right may not be desirable as some of them may break farm-level NPV constraints or others may impose unreasonable costs. Nontheless, ecological improvements do not invariably necessitate high public expenditure (Fig. 4c). For instance, the policy achieving the highest observed connectivity is realized at a moderate-high government cost (though may still break farm-level NPV constraint), far from the most expensive options (Fig. 4c). Conversely, some policies like the one with the lowest observed connectivity, despite incurring substantial costs, yield poor connectivity outcomes, even falling below baseline levels (Fig. 4c), and such inefficiencies are further highlighted in the correlation analysis (Supplementary Fig. 13). This highlights that smart policy design is required to ensure cost-effectiveness and avoid inefficient allocation of resources. Furthermore, the range of policies that are output depends on the weights *w*_*C*_ and *w*_*NPV*_ and the specific goal. For instance, assigning a higher weight to connectivity (*w*_*C*_) shifts the optimization toward policies with stricter mandates or higher incentive payments (e.g., high eco-premiums), ensuring ecological targets are met. Conversely, prioritizing economic stability (*w*_*NPV*_) favors policies that rely on voluntary, lower-cost interventions that minimize disruption to farm operations, potentially at the expense of maximal landscape cohesion.

We then filtered out the policies that satisfied all our constraints (min farm-level NPV difference, low cost, minimum connectivity difference), and examined key policy parameter distributions in top-performing policies (Fig. 4b and Supplementary Fig. 12). We saw that effective designs often involve moderate subsidy levels for intervention establishment and maintenance, with medians for various factors typically ranging from 0.2 to 0.3 within their 0.0–0.5 search spaces. However, the mandate parameter Min. Margin Frac. Adj. Habitat (Mandated minimum fraction of margin adjacent to existing habitats, with a 0.0–0.3 search range) consistently skewed towards lower values, with a median around 0.1 in these successful policies (Fig. 4b and Supplementary Fig. 12). Exploring this further, we visualized specific successful policy archetypes, including parameters like eco-premiums, habitat area payments, and total habitat mandates. These profiles illustrate how different optimization objectives shape the policy levers. For example, a policy targeting good connectivity with maximum NPV and minimal deviation from baseline connectivity tends to employ substantial eco-premiums across multiple crops and moderate habitat maintenance subsidies (Fig. 4d and Supplementary Table 5). In contrast, policies prioritizing minimal cost while achieving good connectivity generally exhibit lower subsidy rates and eco-premiums. Finally, the policy archetype that minimizes the combined difference in connectivity and farm-level NPV relative to the optimized baseline features high-margin maintenance subsidies and habitat payments, but notably lacks mandates.

Therefore, the optimal policy choice depends on the specific goal: minimizing farm-level NPV difference, ensuring low cost, or minimizing connectivity difference. Rather than a single universal solution, the framework identifies a range of viable policies that vary based on the weights *w*_*C*_ and *w*_*NPV*_ and the prioritized objectives. While hybrid instruments often perform well, the ultimate selection requires balancing these competing ecological and economic goals.

## Discussion

This study demonstrates that economically grounded habitat restoration and spatially strategic ecological planning can be combined to transform fragmented agricultural landscapes. We show that while farm-level intensification generates local benefits consistent with empirical gradients^1,2^, it fails to produce landscape-scale connectivity. In contrast, our integrated multi-scale framework identifies that small, strategically placed restorations can increase landscape connectivity substantially without compromising farm viability. The key mechanism driving this result is the nonlinearity of connectivity: a few “stepping stone/hub" patches can bridge large gaps, unlocking network-wide pathways^19,27,44^. These hub locations are ecologically strategic because they reduce effective distances between habitat clusters, enabling movement pathways that would otherwise be absent. This stepping-stone dynamic is a fundamental mechanism governing connectivity in fragmented landscapes^34,41,44^. Importantly, our optimization confirms these gains are economically feasible. By respecting opportunity costs, the model avoids unrealistic land retirement, aligning with calls for conservation that reconciles ecological goals with economic realities^15^.

Our results advance the understanding of how policy design can bridge the gap between farm-level economic decisions and landscape-scale ecological goals. Previous bio-economic modeling has often focused on optimizing land use for ecosystem services at the farm scale^28–30^ or designing cost-effective agri-environmental schemes without explicit spatial connectivity targets^25,38^. Conversely, conservation planning studies have developed sophisticated methods for identifying optimal habitat networks^33,34,44^, yet these frequently abstract away from the economic constraints and decision-making processes of individual landowners. By integrating these dimensions into a single hierarchical framework, we demonstrate that the trade-off between economic and ecological objectives is not fixed but depends critically on the spatial configuration of interventions.

Consistent with literature on policy mechanism design, we find that no single instrument is universally optimal^12^. While spatially targeted payments are theoretically most efficient^25^, our Bayesian policy search reveals that hybrid approaches, combining broad-based stewardship payments with specific adjacency bonuses, can approximate the performance of fully targeted schemes while remaining administratively simpler. This aligns with findings by Verhagen et al.^28^ and others^26,40^, who suggest that successful policy mixes must balance prescription with flexibility. However, our framework goes further by quantifying the specific parameters (e.g., subsidy rates, eco-premiums) required to tip the landscape from a fragmented to a connected state, offering a more operational tool for policy calibration.

Our policy evaluation reveals that the effectiveness of agri-environmental instruments depends critically on the specific prioritization of goals. While hybrid instruments, combining mandatory baselines with targeted incentives, often provide a robust balance, our results show that the optimal policy architecture varies based on the relative weights assigned to ecological connectivity, public cost, and farm-level profitability. This finding highlights the necessity of moving beyond “one-size-fits-all" recommendations toward bespoke policy designs that reflect specific regional or societal objectives. Whether the goal is to maximize connectivity at any cost or to achieve moderate improvements with minimal economic disruption, the framework identifies the precise combination of levers required. While our study focuses on subsidies and mandates, the Bayesian framework is agnostic to the policy mechanism and could be extended to evaluate tradable permits, eco-credits, or auction-based conservation tenders.

A particularly important implication concerns climate resilience. Connectivity enhances the ability of species to track shifting climatic conditions, maintain gene flow, and persist in fragmented landscapes^35^. In agricultural regions where semi-natural habitat is sparse, restoring small but strategically located patches may be essential for enabling range shifts and ecological adaptation. The framework presented here identifies which patches matter most for facilitating these processes, offering planners a structured way to incorporate climate adaptation considerations into land-use decisions. The fact that these patches also tend to be economically feasible to restore strengthens the case for integrating connectivity priorities into mainstream agricultural policy rather than treating them as niche conservation concerns.

The robustness of the results across ecological and economic uncertainties further strengthens the framework's generalizability. Agricultural landscapes vary widely in crop composition, habitat distribution, and management practices, yet many exhibit similar connectivity bottlenecks driven by structural constraints such as the spatial arrangement of fields, natural features, and remnant vegetation^36^. The persistence of keystone patches across sensitivity analyses suggests that landscape geometry itself governs much of the connectivity potential. This echoes empirical studies showing that ecological flows, dispersal, gene flow, and movement are disproportionately shaped by a limited number of structural impediments or conduits, regardless of variation in species traits or environmental conditions^18,37^. Because the model integrates empirical dispersal ranges and spatially explicit habitat maps, it captures these structural features in a way that is broadly transferable to other regions with similar land-use mosaics.

**Fig. 5:**
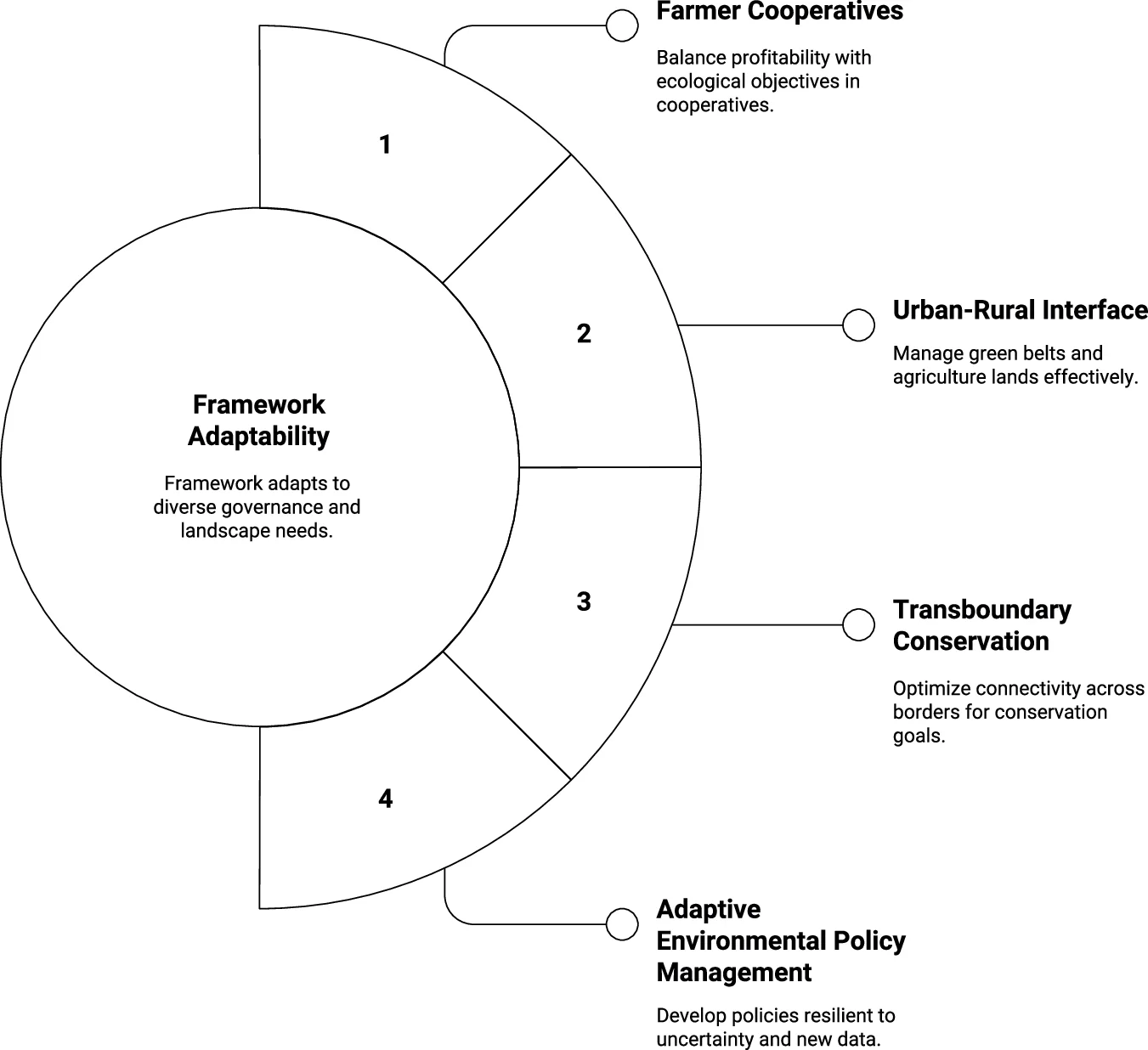
Applications and extensions of the proposed framework. This figure illustrates the framework's versatility for designing tailored, adaptive environmental policies across diverse, complex scenarios. The model’s components can be adapted for various governance structures, such as helping farmer cooperatives align individual member profitability with collective ecological goals, or aiding planners at the urban-rural interface in designing incentives that preserve green belts under development pressure. For large-scale challenges such as transboundary conservation, the framework can be used to harmonize policies across jurisdictions to meet shared environmental targets. A crucial, cross-cutting application is its inherent support for adaptive management.

The adaptability of our framework facilitates tailored policy design across diverse contexts (Fig. 5). Farmer cooperatives could use it to balance individual profits with collective ecological goals, while planners in urban-rural interfaces could design incentives to preserve green belts. Our architecture is generalizable; however, the specific inputs and outputs (e.g., land percentages, subsidy magnitudes) should be modified to be landscape- and context-specific. By linking farm-level decisions, landscape-scale optimization, and policy design, our approach offers a practical pathway for shifting agricultural systems toward multifunctionality and climate resilience.

Despite its strengths, the framework has limitations that suggest opportunities for further development. First, while the EI component captures two major service pathways, pollination and natural pest control, it does not explicitly model other ecosystem functions such as soil fertility, hydrological regulation, or carbon storage, which may also influence profitability and ecological outcomes. Many of these services operate at spatial or temporal scales different from those of pollination and pest suppression, and integrating them could further refine predictions of optimal restoration. Second, the connectivity metric is based on structural connectivity rather than species-specific functional connectivity. While structural measures like the Integral Index of Connectivity are widely used and correlate strongly with movement potential^5,16^, functional connectivity may diverge for species with complex behavioral responses to landscape structure^41^. Therefore, explicit modeling of species movement could refine the ecological implications of restoration priorities. Such extensions may require integrating circuit-theory models or agent-based dispersal simulations^23,29,42,43^.

Third, the model assumes that farms respond deterministically to economic incentives, whereas behavioral studies show that adoption of conservation practices is often influenced by risk preferences, social norms, and perceptions of environmental stewardship^33^. Incorporating behavioral heterogeneity could change the spatial distribution of restoration and may reveal policy designs that are robust across different farmer types. Fourth, the framework captures static connectivity patterns, whereas real-world ecological networks are dynamic. Temporal factors such as habitat maturation, crop rotation, and climate-driven range shifts will inevitably alter connectivity priorities and the location of critical “hubs” over time. For instance, as restored habitats mature, their resistance to movement may decrease, potentially solidifying certain corridors while making others redundant. Similarly, shifting crop patterns could periodically disrupt or create temporary stepping stones. Integrating temporal dynamics into future iterations of the model could help to identify restoration strategies that remain ecologically beneficial over longer time horizons, particularly under climate change scenarios that require dynamic movement pathways for species adaptation^13,35,38,46^.

## Methods

### Overall framework and study region

Our modeling framework integrates farm-level economic decisions, spatial ecological processes, and policy design into a unified hierarchical system calibrated to a real agricultural landscape. Each component reflects the scale at which ecological functions operate, farmers make decisions, and policies exert influence, allowing ecological realism and economic behavior to shape outcomes simultaneously. By linking ecological intensification with connectivity optimization and policy search in a sequential manner, the framework ensures that ecological improvements remain feasible for producers and that policy outcomes remain grounded in biophysical and economic realities.

The study region is a mixed-farming landscape in Manitoba, Canada (Fig. 1b) that reflects the structure of many agricultural regions, characterized by crop mosaics, fragmented semi-natural habitats, and privately owned parcels. High-resolution land-cover layers were assembled from remote sensing and classification products identifying cropland, woody patches, herbaceous habitat, and non-agricultural surfaces^47,48^. Parcel boundaries were integrated with crop distribution data to construct farm-level decision units (Supplementary Fig. 4). Ecological layers representing pollinator foraging ranges, natural enemy dispersal distances, and habitat influence zones were derived from published empirical studies documenting the spatial decay of service provision in agricultural systems^4,9–11^. These layers were rasterized to a common resolution to ensure consistent treatment of ecological, economic, and policy components^1^. Ecological influence layers were constructed using distance-decay kernels derived from empirically observed dispersal distances for pollinators and natural enemies^4,9,15,16^. For each spatial unit, influence weights quantify the expected contribution of nearby habitat or intervention sites to ecosystem service provision, with contributions declining as a function of distance (see Supplementary Eqs. 1, 2 and Supplementary Table 1). These layers are used to calculate ecosystem service contributions in the EI model and to evaluate habitat network structure in the connectivity optimization.

Refer to Table 1 for details of notations used in the framework.

**Table 1:** Notation used in the hierarchical optimization framework

Symbol	Description
*i*	Index for farms
*t*	Index for time (years)
*h*_*i*_	Vector of habitat restoration decisions for farm *i*
NPV_*i*_	Net Present Value of farm *i*
*Π*_*i*,*t*_	Annual profit of farm *i* at time *t*
*r*	Discount rate
*T*	Time horizon
IIC	Integral Index of Connectivity
*H*	Landscape-wide habitat configuration
EI_NPV_*i*_	Baseline NPV for farm *i* under ecological intensification
*Δ*	Maximum allowable economic loss (tolerance parameter)
*θ*	Vector of policy parameters
*α*	Weighting factor in policy objective function
IIC^*^	Target connectivity benchmark from EC optimization

### Ecological intensification (EI) model

The first component of the framework models ecological intensification (EI) at the scale of individual farms. It infers economically efficient habitat shares under farmer decision-making. We assume crop choices are fixed based on current land use, focusing the optimization on the margin and habitat configuration within this established cropping system. Because field margins and internal habitats are not directly observable in the baseline landscape, EI provides the farm-level reference point (NPV_*i*_) and economically acceptable deviation (*Δ*) used in the connectivity optimization. This ensures that Step 2 does not compare ecological designs to unrealistic profit levels.

For each farm *i*, the model identifies the vector of habitat restoration decisions *h*_*i*_ that maximizes net present value (NPV) of profits over a time horizon *T*. The profit function incorporates revenues, input costs, ecological service gains, and opportunity costs associated with converting cropland to habitat. Formally, the farm solves:

1 $$\max \mathop{\sum }\limits_{i\in {I}_{ag}}NP{V}_{i}+\mathop{\sum }\limits_{i\in {I}_{hab}}NP{V}_{i}-{\rm{\lambda }}\mathop{\sum }\limits_{i\in {I}_{ag}}(\mathop{\mathop{m}\limits^{2}}\limits_{i}+\mathop{\mathop{h}\limits^{2}}\limits_{i})$$

where *I*_*ag*_ is the set of agricultural plots, and *I*_*hab*_ is the set of existing habitat plots within the farm. *NPV*_*i*_ represents the Net Present Value calculated for plot *i*, incorporating costs, revenues, and the ecosystem service benefits (pollination and pest control) generated by interventions. The decision variables for each agricultural plot *i* are: *m*_*i*_, the fraction of plot *i*’s area allocated to enhanced field margins (0≤*m*_*i*_≤1), and *h*_*i*_, the fraction of plot *i*’s area converted to habitat (0≤*h*_*i*_≤1). The final term is a penalty function with coefficient *λ* applied to the squared decision variables, primarily aiding numerical stability and acting as a form of regularization. Without this term, the optimization landscape led to unrealistic and extreme solutions. It is important to note that this farm-level optimization assumes that farmers make decisions based on the benefits they capture within their own property boundaries, accounting for ecological service spillovers from neighboring farms.

The NPV for each plot *i* is calculated based on the discounted cash flows over the time horizon *T*. For Agricultural Plots (*i* ∈ *I*_*ag*_), the NPV calculation involves initial implementation costs and annual net cash flows (revenue minus maintenance costs and yield loss from habitat conversion), discounted back to the present:

2 $$NP{V}_{i}=-{C}_{impl,i}+\mathop{\sum }\limits_{t=1}^{T}\frac{{R}_{i,t}-{C}_{maint,i}}{{(1+r)}^{t}}$$

where *C*_*impl*,*i*_ is the implementation cost, *R*_*i*,*t*_ is the revenue, *C*_*maint*,*i*_ is the maintenance cost, and *r* is the annual discount rate reflecting the time value of money. The yield per unit area is enhanced by pollination and pest control services originating from interventions on plot *i* and its neighbors *j* ∈ *N*(*i*):

3 $${Y}_{i,t}={Y}_{base,i}\cdot (1+{P}_{i,t}+{S}_{i,t})$$

where *Y*_*base*,*i*_ is the baseline yield, *P*_*i*,*t*_ is the fractional yield increase from pollination, and *S*_*i*,*t*_ is the fractional yield increase from pest suppression at time *t*. Ecosystem service contributions are modeled as proportional modifiers to baseline crop yield, where total yield is calculated as the baseline yield multiplied by an enhancement factor (1 + *P*_*i*,*t*_ + *S*_*i*,*t*_) representing the combined benefits of pollination and pest control. This enhanced yield alters Net Present Value (NPV) over the planning horizon (see Supplementary Eqs. 1, 2 and Supplementary Table 1 for details). Empirical studies have demonstrated that pollination and biological pest control can enhance crop productivity and profitability, particularly when habitats are located within effective dispersal distances^7,10,11,35^.

Pollination (*P*_*i*,*t*_) and Pest Suppression (*S*_*i*,*t*_) effects accumulate over time and decay with distance. They are calculated by summing the contributions from plot *i* itself and its neighbors *j* ∈ *N*(*i*) (including other agricultural plots and existing habitat plots). The effect of an intervention (margin *m*_*j*_ or habitat *h*_*j*_) on plot *j* contributing to plot *i* at time *t* depends on the distance *d*_*ij*_. Detailed mathematical formulations for *P*_*i*,*t*_ and *S*_*i*,*t*_, including decay functions and crop-specific parameters, are provided in the Supplementary Information (Supplementary Figs. 6 and 7; Supplementary Table 1).

### Ecological connectivity (EC) model

The second component shifts from farm-level decisions to landscape-scale ecological structure by identifying where additional habitat restoration would most improve connectivity. Connectivity was measured using the Integral Index of Connectivity (IIC), a graph-theoretic metric widely used in landscape ecology because it captures both habitat availability and the configuration of dispersal pathways^15,16,27,34,44,49,50^.

The connectivity optimization seeks the habitat configuration *H* that maximizes IIC subject to farm-level economic constraints. Specifically, the model ensures that no farm’s NPV falls below an acceptable threshold relative to its EI baseline:

4 $${N}_{f,new}\ge (1-\Delta ){N}_{f,base}$$

where *N*_*f*,*new*_ and *N*_*f*,*base*_ are the farm’s Net Present Value in the new and baseline configurations, respectively, and Δ represents the maximum allowable economic loss (tolerance parameter). This constraint addresses the requirement that connectivity improvements must be realistic for individual operations, as farms bear the opportunity costs of restoration^28,29,46^. While aggregate landscape-level constraints are conceivable, farm-level constraints ensure that no single producer is disproportionately burdened, which is critical for the political feasibility of voluntary agri-environmental schemes.

The primary objective is to maximize a landscape connectivity metric, *Z*:

5 $$\,{\rm{Maximize}}\,Z=\mathop{\sum }\limits_{i\in P}{s}_{i}{x}_{i}+\mathop{\sum }\limits_{(i,j)\in A}{w}_{ij}{y}_{ij}$$

Where *x*_*i*_ is a binary decision variable (1 if piece *i* is selected), *s*_*i*_ is the intrinsic ecological score of piece *i*, *y*_*ij*_ is a binary decision variable (1 if both piece *i* and piece *j* are selected and are adjacent), and *w*_*ij*_ is the weight of the connection. *P* represents the set of all potential habitat spatial units, and *A* represents the set of all adjacent unit pairs in the landscape. The intrinsic ecological score *s*_*i*_ represents the contribution of spatial unit *i* to overall habitat availability and ecological function. Connectivity weights *w*_*ij*_ represent the strength of ecological connections between spatial units, based on spatial adjacency and dispersal potential. This formulation follows established graph-theoretic approaches to ecological connectivity modeling, where habitat availability and spatial configuration jointly determine connectivity outcomes^34,44,50^.

The optimization problem is spatially explicit and nonlinear. It requires mixed-integer nonlinear programming (MINLP) to capture discrete restoration decisions and the nonlinear connectivity function. The computational challenges associated with over 10^5^ spatial cells and thousands of potential restoration locations necessitated dividing the landscape into configuration units. These units were constructed to preserve ecological influence zones based on empirical dispersal thresholds^4,10^. Sensitivity analyses confirmed that connectivity outcomes were robust to alternative unit definitions, ensuring that partitioning did not introduce artificial patterns (Supplementary Fig. 5 and Supplementary Table 2). The optimal habitat configuration resulting from this landscape-scale optimization is denoted as H^*^ and serves as the ecological target for the subsequent policy search. We also define a “repositioning” baseline, which refers to reallocating intervention locations while holding total intervention area constant, allowing isolation of spatial configuration effects from intervention quantity.

### Bayesian policy optimization (BO)

The third component of the modeling framework evaluates which agri-environmental policy instruments are capable of inducing restoration patterns that approximate the connectivity benchmark derived from the optimization. Because farmers respond to policy incentives rather than to global ecological objectives, policy outcomes must be simulated by re-solving the EI problem under modified profit structures.

The policy evaluation is framed as a black-box optimization problem. The goal is to find a policy *θ* (Supplementary Table 3) that induces landscape connectivity as close as possible to the optimal connectivity benchmark H^*^ identified in Step 2, while maintaining economic acceptability. The policy parameter vector *θ* includes establishment subsidies, maintenance payments, eco-premiums, and habitat requirements (see Supplementary Table 3). The optimization seeks to identify the specific combination of these parameters that best satisfies the weighted objective function. Formally, each candidate policy *θ* is assessed using an objective function that minimizes the deviation from the optimized IIC and the deviation from EI-based profitability:

6 $${O}_{BO}(\theta )=\frac{1}{{N}_{S}}\mathop{\sum }\limits_{k=1}^{{N}_{S}}\left({w}_{C}\cdot \left|{C}_{L,k}^{policy}(\theta )-{C}_{L,k}^{target}\right|+{w}_{NPV}\cdot \left(\frac{1}{| {F}_{k}| }\mathop{\sum }\limits_{f\in {F}_{k}}\left|NP{V}_{f,k}^{policy}(\theta )-NP{V}_{f,k}^{target}\right|\right)\right)$$

where ${C}_{L,k}^{policy}(\theta )$ and $NP{V}_{f,k}^{policy}(\theta )$ are the landscape connectivity and farm *f*’s NPV under policy *θ* in configuration iteration *k*, while ${C}_{L,k}^{target}$ and $NP{V}_{f,k}^{target}$ are the corresponding values under the target benchmark (H^*^, EI baseline). The terms *w*_*C*_ and *w*_*NPV*_ are weights determining the relative importance of the ecological (connectivity) and economic (NPV) objectives, respectively; *N*_*S*_ is the number of samples, and *F*_*k*_ is the set of farms in configuration *k*. This formulation reflects the dual nature of agri-environmental policy design, which must align ecological goals with producer incentives^35,36^. By minimizing the deviation from the theoretical optimum, the search identifies policies that can realistically achieve the “best-case” ecological scenario identified in Step 2. This formulation effectively maximizes connectivity within the feasible economic region defined by the policy parameters, ensuring that the resulting landscape approaches the theoretical benchmark as closely as possible given the constraints of voluntary adoption.

Exploring this policy space exhaustively is computationally prohibitive because each policy evaluation triggers EI optimization across all farms, reconstruction of habitat distribution, and recomputation of landscape connectivity. To address this challenge, Bayesian optimization was used as an efficient search strategy (Supplementary Table 4). Bayesian optimization employs a probabilistic surrogate model, here, a Gaussian process, to approximate the expensive objective function and an acquisition function to select new policies that are likely to yield improvement^45^. This approach has been widely adopted for computationally expensive optimization in engineering, economics, and environmental modeling because it requires far fewer evaluations than grid search or evolutionary algorithms^38,45,51,52^.

### Model validation and robustness testing

To ensure the reliability of the framework, we conducted validation and robustness checks at each stage. For the EI model, we verified that the restoration levels and yield-service elasticities fell within ranges reported in global empirical syntheses^2,4^, confirming that the parameterized ecological kernels produce realistic farm-level behavior. For the connectivity optimization, we assessed the structural properties of the generated networks against established ecological theory. The emergence of nonlinear connectivity gains and the identification of keystone “stepping stone/hub" patches are consistent with graph-theoretic predictions for fragmented landscapes^27,34^. Finally, sensitivity tests varying key economic and ecological parameters confirmed that the identity of high-influence nodes and the superiority of certain policy instruments remain stable, underscoring that connectivity is governed primarily by the structural geometry of the landscape rather than specific parameter values. We also validated the model using synthetic data (Supplementary Figs. 14–17).

## Supplementary information

Supplementary Information

## Data Availability

The data that support the findings of this study are publicly available to download and are referenced in the bibliography. Refer to the Methods section for more details. All project-specific data-generating scripts are made available in the GitHub repository.

## Code availability

The code repository for this project, including data processing, optimization models, simulation workflows, and visualization scripts, is available in the GitHub repository^53^.
