# Supplementary material for: An integrated multi-scale framework for economically feasible landscape connectivity in working agricultural systems
Source: NPJ Sustain Agric. 2026 Oct 30;4(1):89. doi: 10.1038/s44264-026-00166-5 (PMC13722584; doi:10.1038/s44264-026-00166-5)
Supplement: Supplementary file 1 [file 44264_2026_166_MOESM1_ESM.pdf]

Supplementary Information: Bridging Farm Economics and Landscape Ecology for Global Sustainability through Hierarchical and Bayesian Optimization

Detailed Model Formulation

Ecological Intensification (EI) Model Details

The pollination ($P_{i,t}$) and pest suppression ($S_{i,t}$) effects are calculated as follows:

Pollination Effect ($P_{i,t}$):

$$P_{i,t} = \sum_{j \in N(i) \cup \{i\}} \left[(\alpha m_j e^{-\beta d_{ij}})(1 - e^{-\gamma t}) + (\alpha_h h_j e^{-\beta_h d_{ij}})(1 - e^{-\gamma_h t}) \right] + \sum_{k \in N_{hab}(i)} (\alpha_h e^{-\beta_h d_{ik}})(1 - e^{-\gamma_h t}) \quad (1)$$

Pest Suppression Effect ($S_{i,t}$):

$$S_{i,t} = \sum_{j \in N(i) \cup \{i\}} \left[(\delta m_j e^{-\epsilon d_{ij}})(1 - e^{-\zeta t}) + (\delta_h h_j e^{-\epsilon_h d_{ij}})(1 - e^{-\zeta_h t}) \right] + \sum_{k \in N_{hab}(i)} (\delta_h e^{-\epsilon_h d_{ik}})(1 - e^{-\zeta_h t}) \quad (2)$$

Here, α, β, γ and δ, ϵ, ζ are the strength, distance decay, and time accumulation parameters for pollination and pest control from margins, respectively. Parameters with subscript h ($\alpha_h, \beta_h, ...\zeta_h$) represent the corresponding effects from habitat (converted or existing). $N_{hab}(i)$ is the set of neighboring plots that are existing habitats. All parameters are specific to the crop type on plot i. The time accumulation factors $(1 - e^{-(\cdot)t})$ are precomputed for efficiency.

EI Model Analysis

While the EI model optimizes purely for the NPV summed across all farm plots, the underlying economic logic creates emergent, non-random spatial patterns in the recommended interventions. The bar chart summarizing Pearson correlation coefficients (Supplementary Fig. 1) quantifies the strength and direction of relationships between key plot factors and the optimal levels of margin and habitat interventions. The analysis revealed weak relationships between plot characteristics and the optimal intervention strategies selected by the model. Notably, plot yield exhibited a slight negative correlation ($r = -0.12$) with the decision to implement margins (Supplementary Fig. 1), however, this correlation seems superficial according to the scatter plot (Supplementary Fig. 2a). Geometric factors display weak negative correlations with habitat conversion ($r \approx -0.11$ to -0.15). This suggests that larger or more geometrically complex plots are not favoured for habitat conversion in this optimization (Supplementary Fig. 1, Supplementary Fig. 2b). While costs scale with area in the model, the benefits generated are influenced by the intervention fraction and proximity to neighbours. The negative correlation might arise if the increasing costs on larger plots slightly outweigh the benefits, or if smaller plots are strategically located such that their conversion offers higher marginal NPV gains to the overall farm system. Moreover, the hypothesis that habitat interventions are directed to where marginal gains are highest (further from existing habitats) is not strongly supported by these outcomes (Supplementary Fig. 1). This could be a result of multiple habitats existing in the landscape in close proximity, thereby diluting the effect of distance to existing habitat.

Model Parameters and Sensitivity

A key strength of the model is its explicit incorporation of spatial, temporal, and crop-specific dynamics influencing the provision of pollination and pest control services generated by interventions. The benefits from new margins or habitats accrue over time, reflecting ecological processes like vegetation establishment or beneficial insect population growth. This is modeled via an exponential saturation function, where the parameter γ (or ζ for pest control) dictates the rate of maturation. As illustrated in Supplementary Fig. 6 and specifically for different crops in Supplementary Fig. 7 (left panel), higher γ values lead to faster attainment of the maximum potential effect level. Ecosystem service effects diminish with distance from the source intervention. The model captures this using an exponential decay function, where β (or ϵ) controls the decay rate. Plots receive benefits from their own interventions and distance-discounted benefits from neighbors. Supplementary Fig. 6 shows the general sensitivity to the decay parameter, while Supplementary Fig. 7 illustrates how this reach varies depending on the crop, service, time, and intervention type.

The model recognizes that different crops exhibit varying dependencies on pollination and benefit differently from pest control. Crucially, the parameters governing the magnitude, temporal dynamics, and spatial decay of these services are crop-specific. Supplementary Fig. 7 explicitly visualizes this, demonstrating how the "Effect Level" for both pollination and pest control, generated by either margins or habitat conversion, varies significantly between crop groups (e.g., Canola/Rapeseed showing higher pollination benefits than Soybeans or Cereals).

Synthetic Data Experiments

The weights used for this random assignment were intended to reflect typical distributions of these categories in the Canadian annual crop inventory (CACI) of 2022. Additionally, a numerical yield value was randomly assigned to each plot, drawn from a distribution as seen in the CACI for the crop in the plot. The final output for each synthetic farm consisted of a GeoJSON FeatureCollection containing the geometric definitions (polygons) and assigned properties (type, label, yield) for all its constituent plots. This process resulted in, for each of the 500 configurations, a structured dataset of synthetic farms composed of multiple labelled plots mimicking real-world agricultural field heterogeneity. See Supplementary Fig. 14-17. All project specific data generating scripts are made available in the GitHub repository.

Supplementary Tables

Table 1: Parameters used in the farm-level Ecological Intensification (EI) model.

Parameter Group	Parameter Definition	Symbol	Value(s)	Unit
**Crop-Specific**				
Spring wheat	Margin Pollination Strength	α	0.05	Unitless
	Margin Pollination Decay	β	0.01	m^{-1}
	Margin Pollination Time	γ	0.2	yr^{-1}
	Margin Pest Strength	δ	0.05	Unitless
	Margin Pest Decay	ϵ	0.01	m^{-1}
	Margin Pest Time	ζ	0.2	yr^{-1}
	Habitat Pollination Strength	α_h	0.05	Unitless
	Habitat Pollination Decay	β_h	0.005	m^{-1}
	Habitat Pollination Time	γ_h	0.2	yr^{-1}
	Habitat Pest Strength	δ_h	0.05	Unitless
	Habitat Pest Decay	ϵ_h	0.005	m^{-1}
	Habitat Pest Time	ζ_h	0.2	yr^{-1}
	Price	p_c	200	USD/Tonne

Parameter Group	Parameter Definition	Symbol	Value(s)	Unit
Barley	(Same params as Wheat)			
	Price	p_c	120	USD/Tonne
Canola/rapeseed	Margin Pollination Strength	α	0.20	Unitless
	Margin Pest Strength	δ	0.10	Unitless
	(Other params same)			
	Price	p_c	1100	USD/Tonne
Corn	(Same params as Wheat)			
	Price	p_c	190	USD/Tonne
Oats	(Same params as Wheat)			
	Price	p_c	95	USD/Tonne
Soybeans	Margin Pollination Strength	α	0.10	Unitless
	Margin Pest Strength	δ	0.10	Unitless
	(Other params same)			
	Price	p_c	370	USD/Tonne
**General Economic**	Discount Rate	r	0.05	Unitless
	Time Horizon	T	20	Years
**Costs**	Margin Implementation	$C_{m,impl}$	400	USD/ha
	Margin Maintenance	$C_{m,maint}$	60	USD/ha/yr
	Habitat Implementation	$C_{h,impl}$	300	USD/ha
	Habitat Maintenance	$C_{h,maint}$	70	USD/ha/yr
	Existing Habitat Maint.	$C_{exist,hab}$	0	USD/ha/yr
	Baseline Ag. Maint.	$C_{ag,maint}$	100	USD/ha/yr

Table 2: Hyperparameters used in the landscape-level EC model.

Parameter	Symbol	Description	Value	Justification
Boundary segments	S_b	Segments per plot	4	Balance flexibility/complexity
Interior cells	S_c	Cells per plot	4	Balance flexibility/complexity
Adjacency dist	d_{adj}	Distance for connection	0.0 m	Strict structural connectivity
Connectivity	IIC	Integral Index of Connectivity	-	Robust metric
Area-Length	α_{AL}	Interaction factor	10^{-9}	Minimize mixed terms
Max NPV loss	Δ	Max allowable loss ratio	0.2	Economic feasibility
Neighborhood	d_{neib}	Dist. for yield effects	1000 m	Ecological scale
Tolerance	-	Solver optimality gap	10^{-6}	Standard precision
Margin weight	w_m	Weight in score	50	Prioritize linear features

Table 3: Ranges of the Policy Parameter Search Space.

Policy Parameter Description	Symbol	Range	Units
Subsidy: margin establishment adj. to habitat	$s_{est,m}$	[0.0, 0.5]	Dimensionless
Subsidy: habitat establishment adj. to habitat	$s_{est,h}$	[0.0, 0.5]	Dimensionless
Subsidy: margin maintenance	$s_{maint,m}$	[0.0, 0.5]	Dimensionless
Subsidy: habitat maintenance	$s_{maint,h}$	[0.0, 0.5]	Dimensionless
Payment per ha for habitat	p_{ha}	[0, 150]	Currency/ha
Mandate: min habitat area per farm	m_{area}	[0, 10]	ha
Mandate: min margin frac adj. to habitat	m_{frac}	[0.0, 0.3]	Dimensionless
Eco-premium factor	e_c	[1.0, 1.3]	Dimensionless

Table 4: Bayesian Optimization (BO) Hyperparameter Configuration.

Hyperparameter	Symbol	Value	Justification
Connectivity Weight	w_C	0.5	Prioritize ecological goal
NPV Weight	w_{NPV}	0.5	NPV is a constraint/outcome
Configuration Samples	N_S	20	Robustness across landscapes
BO Initial Points	N_{init}	15	Initial exploration
BO Total Calls	N_{calls}	100	Computational budget

Supplementary Figures

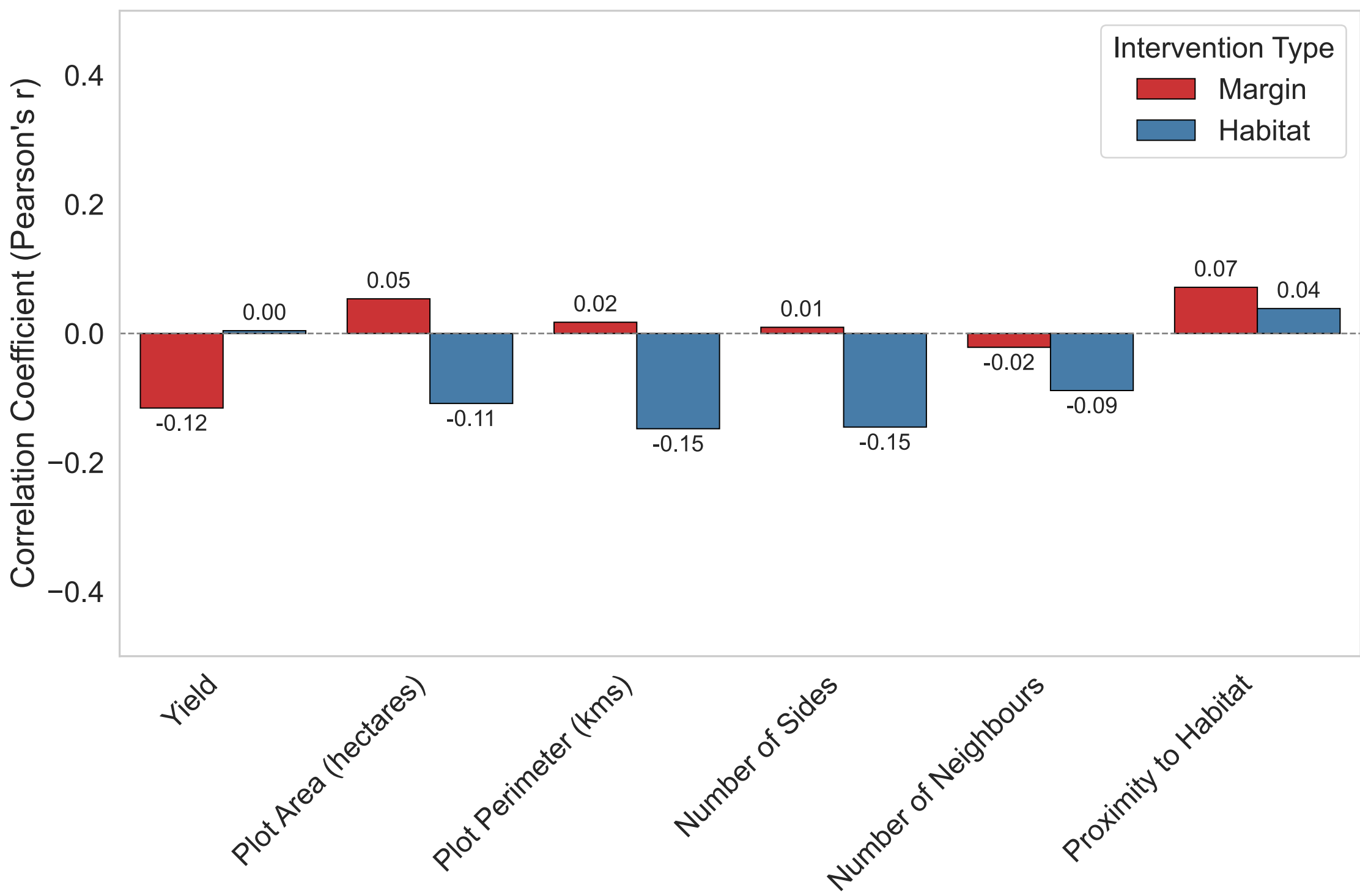

Fig. 1 Pearson correlation coefficients between key plot factors and the optimal levels of margin and habitat interventions. Shown for distance threshold of 1000 m and penalty coefficient of 1e3. The analysis revealed weak relationships between plot characteristics and the optimal intervention strategies selected by the model.

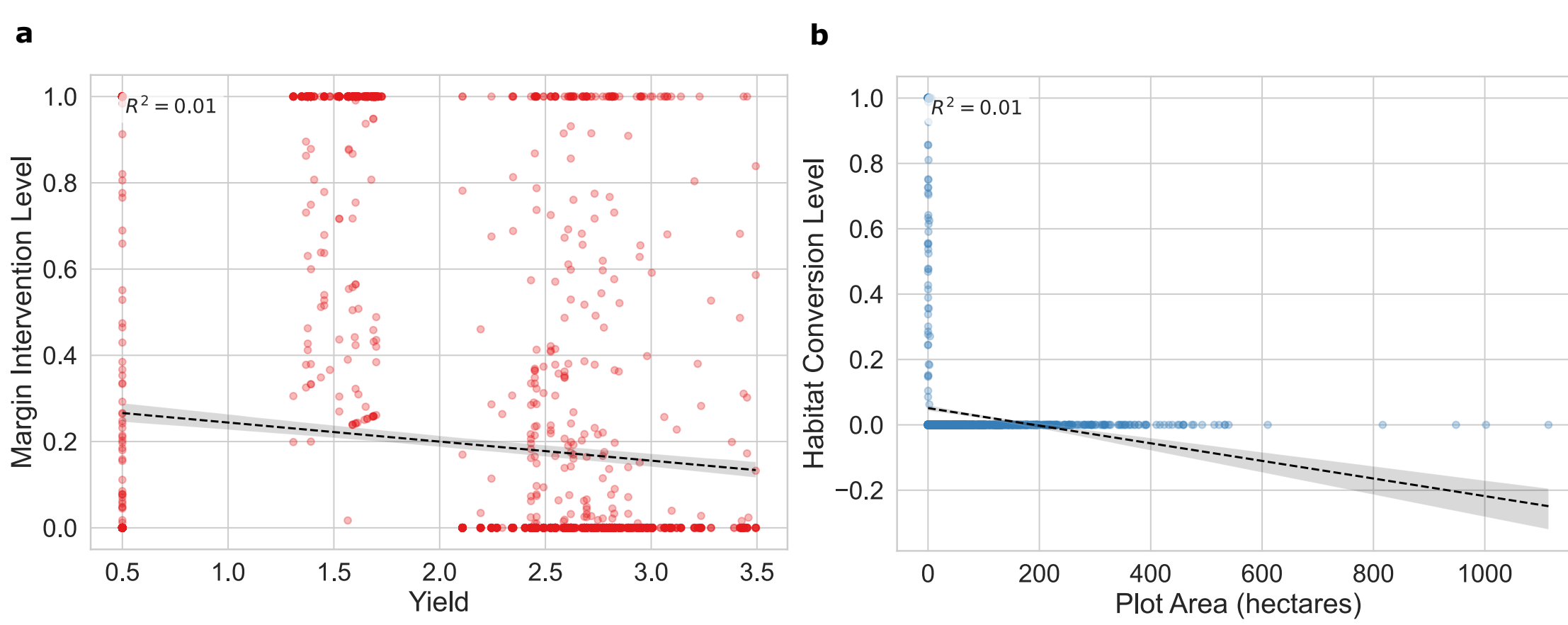

Fig. 2 Scatter plots for intervention levels and selected parameters. a) Scatter plot of margin intervention level and plot yield. The effect of yield appears to be not significant. b) Scatter plot of habitat conversion level and plot area. Smaller plots are strongly preferred for habitat conversion.

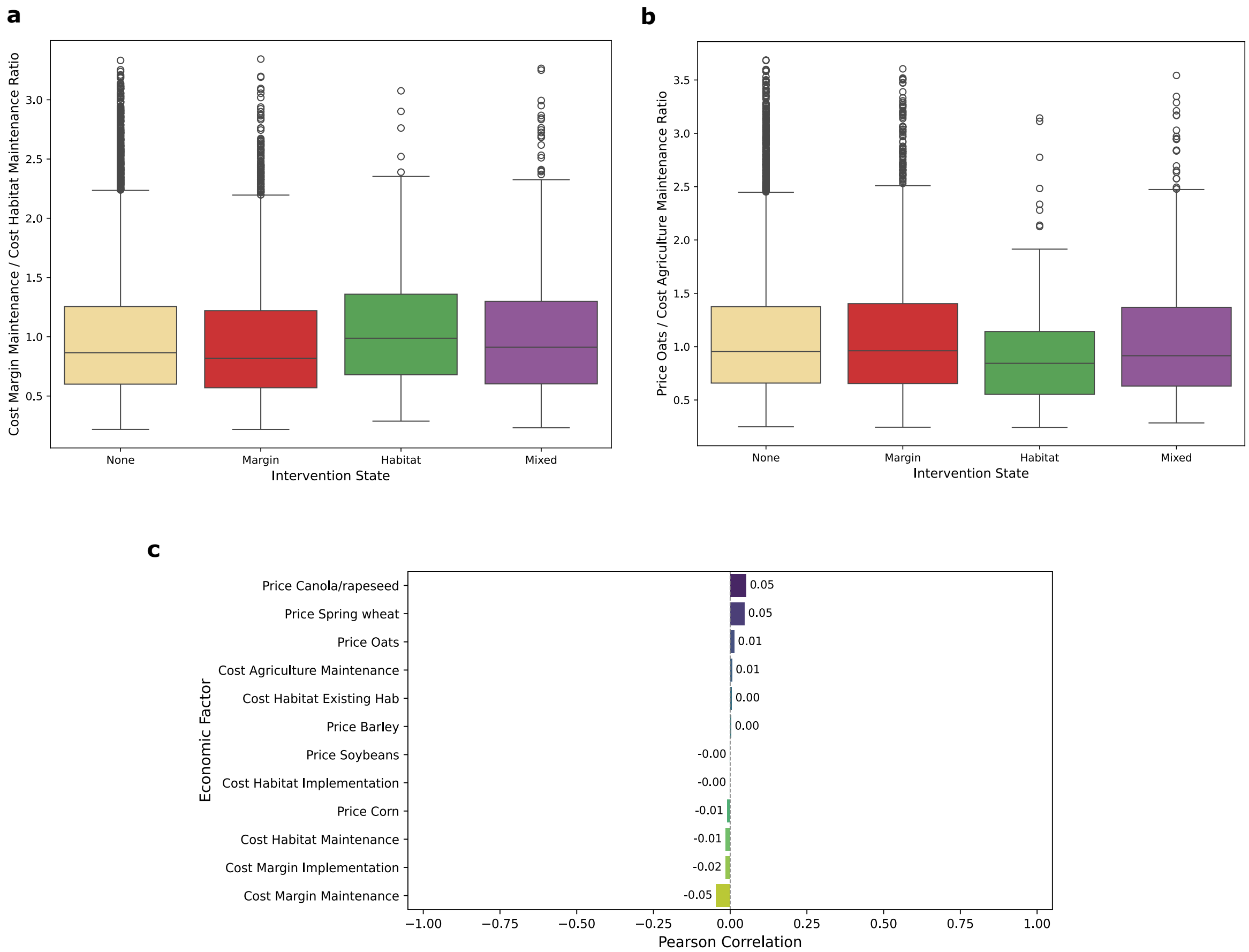

Fig. 3 Sensitivity to costs and prices. a) A box plot of intervention states as a function of the ratio of margin maintenance and habitat maintenance costs. b) A box plot of intervention states as a function of the ratio of price of Oats and agricultural maintenance costs. c) Sensitivity of margin intervention levels to crop prices and costs.

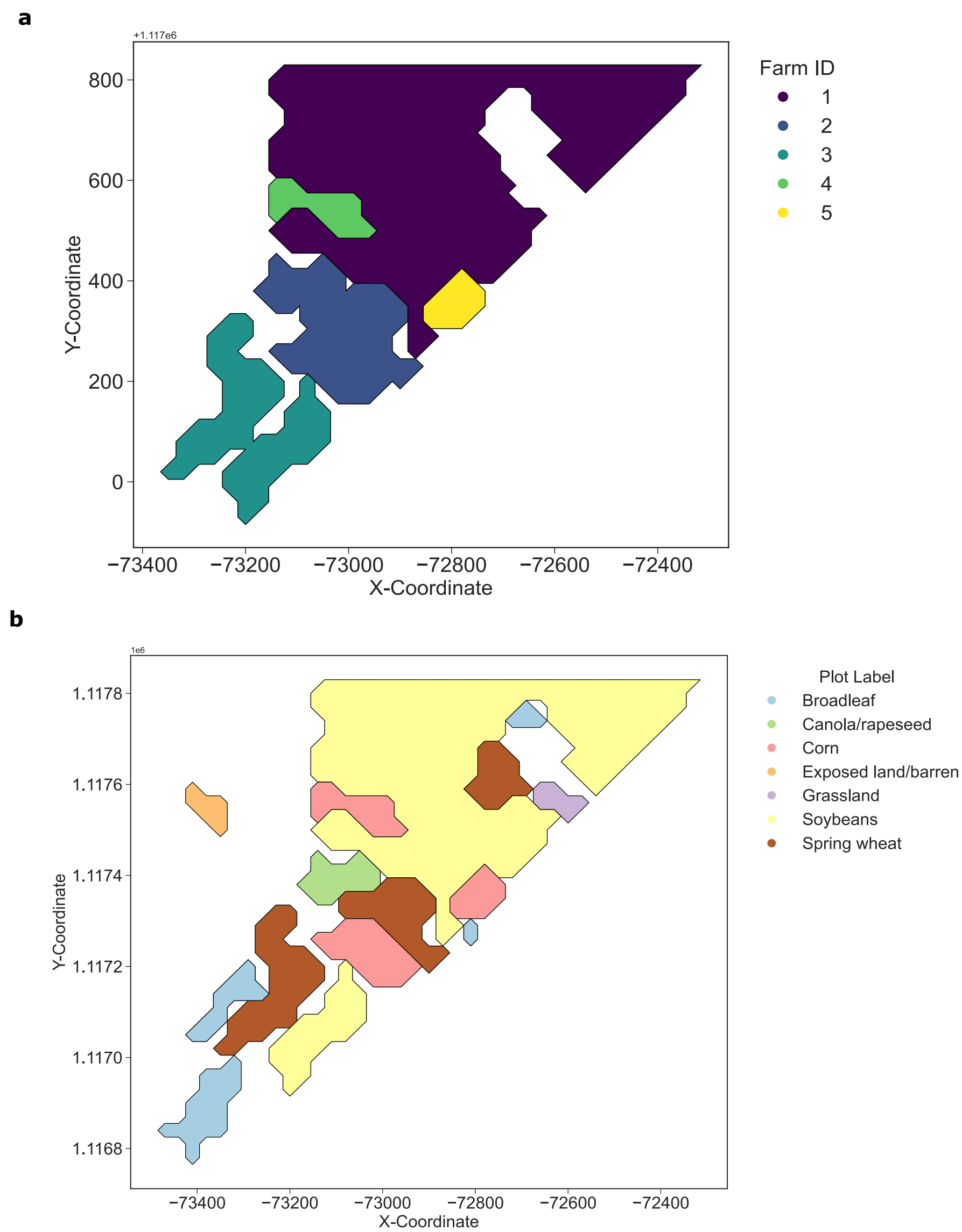

Fig. 4 A selected configuration. a) Farms in the configuration. b) Plots in the configuration.

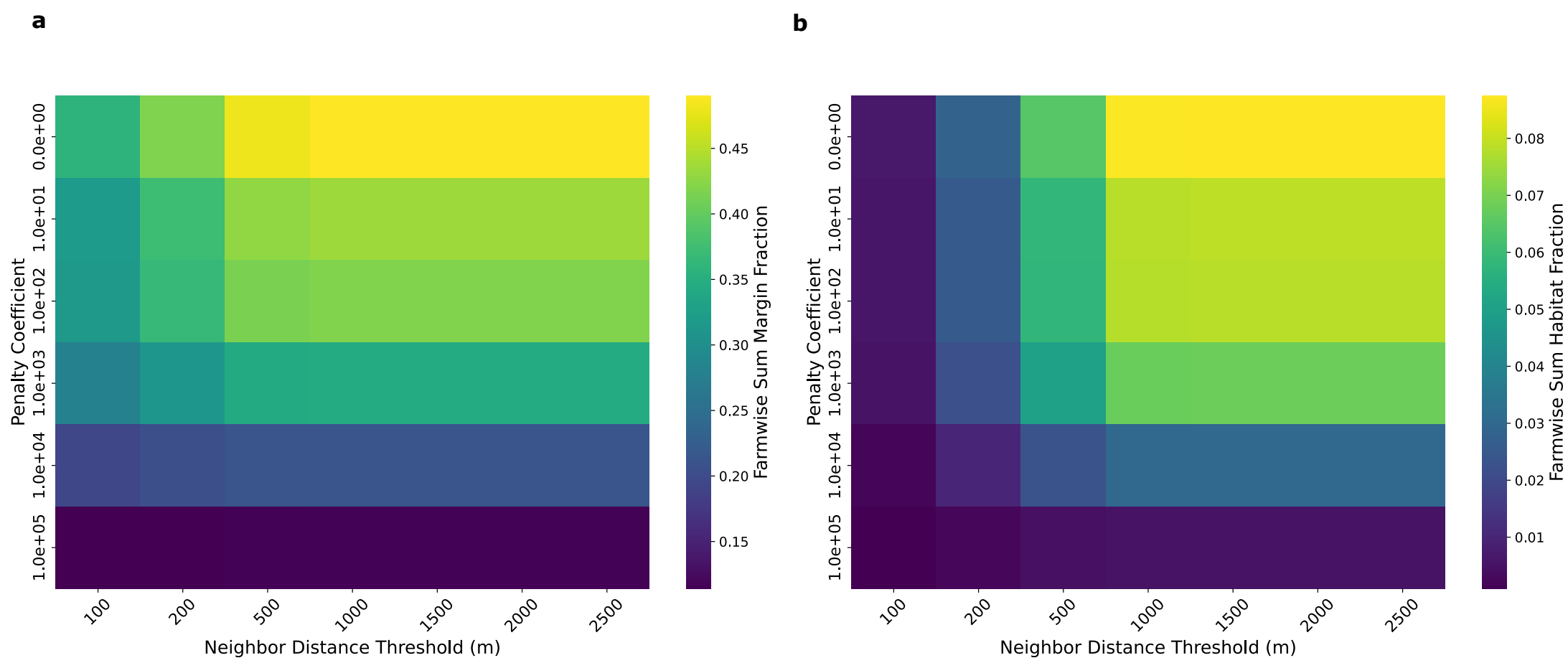

Fig. 5 Heatmap of farmwise sum of intervention values with respect to the penalty coefficient and neighbour distance threshold. a) For margin interventions. b) For habitat conversions. For small values of distance threshold, habitat allocation is extremely low. Therefore we set this threshold to 1000 m. We also observe that for small values of the penalty coefficient margin interventions always take extreme values of either 0 or 1. Therefore, we set the penalty coefficient to 1e3, to get a good spread of fractional interventions as well as achieve regularization.

Table 5 Policy instrument parameters for specific successful policy archetype. These include closest connectivity to baseline, closest connectivity (+-100) and maximum NPV, minimum connectivity difference with baseline per farm, closest connectivity (+-100) and minimum cost, minimum connectivity difference with baseline per farm (+-100) and minimum cost, minimum combined (connectivity+NPV) difference with baseline per farm.

Feature	Policy 14	Policy 12	Policy 8	Policy 1
adj_hab_factor_margin	0	0.0007	0.1869	0.1408
adj_hab_factor_habitat	0.4986	0.0238	0.1173	0
maint_subsidy_factor_margin	0	0.0827	0.4940	0.5
maint_subsidy_factor_habitat	0.4225	0	0.3830	0.0037
hab_per_ha	112.89	147.87	116.55	145.32
min_total_hab_area	0.5554	0.4937	0.2798	0
min_margin_frac_adj_hab	0.2746	0.2892	0.0522	0
eco_premium_factor_Barley	1.0825	1.0367	1.0462	1.2613
eco_premium_factor_Canola	1.2443	1.1628	1.0231	1
eco_premium_factor_Corn	1.0359	1.3	1.2670	1
eco_premium_factor_Oats	1.2225	1.0102	1.2251	1
eco_premium_factor_Soybeans	1	1.3	1.2080	1
eco_premium_factor_Wheat	1.3	1.2745	1.1535	1.0031
avg_conn	7229.61	7261.60	7035.70	6820.27
avg_policy_cost	406973	364486	159560	21254
avg_farm_npv	627763	596349	475337	397511
avg_conn_diff	1708.41	1720.27	1803.65	1863.15
avg_npv_diff	376874	340285	203276	115288
conn_abs_diff_from_baseline	19.52	51.50	174.39	389.82

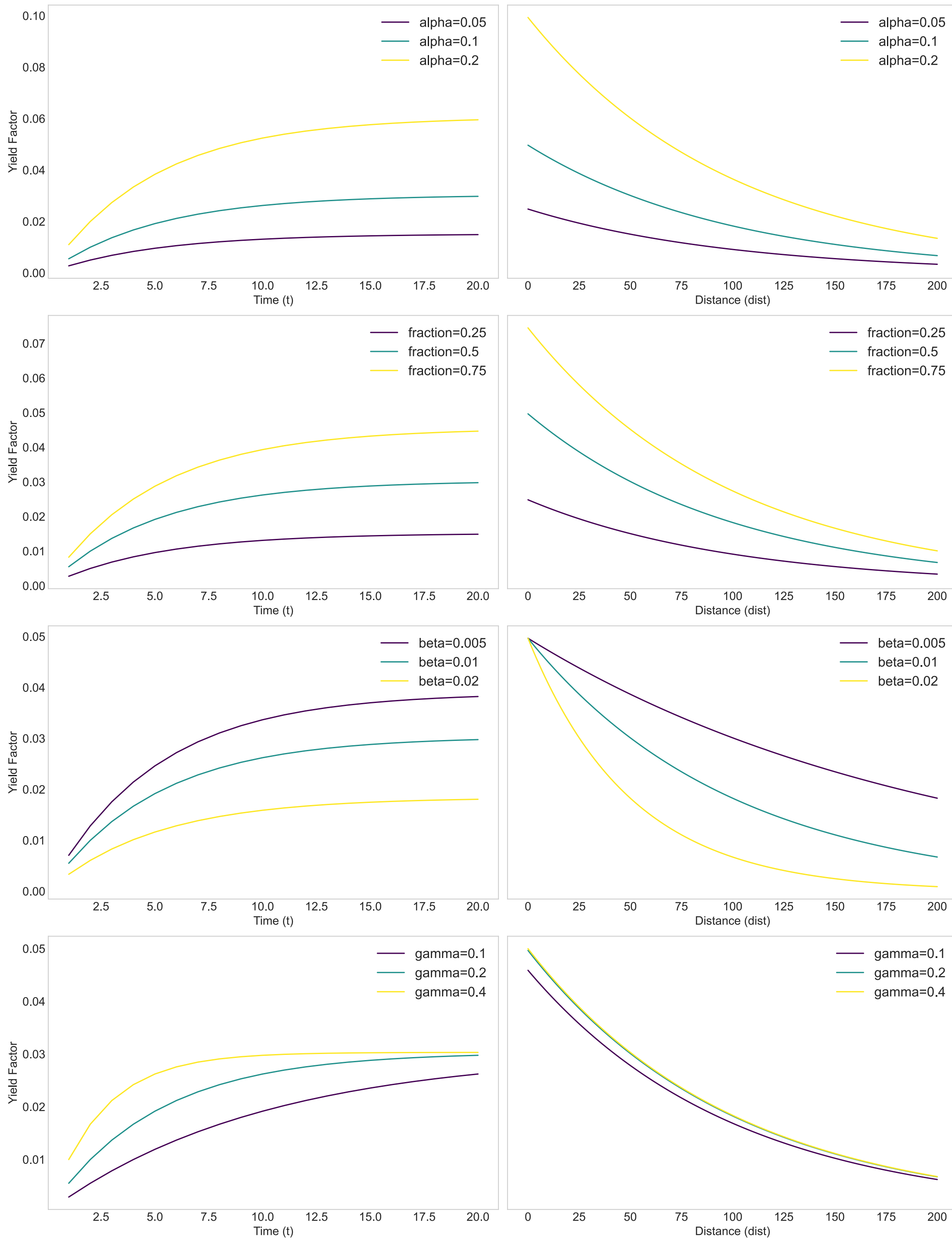

Fig. 6 Yield factor as a function of parameters. Yield factor is the multiplying factor that enhances yield as a result of ecosystem services. Left panel shows behavior with time, and right panel shows behavior with distance.

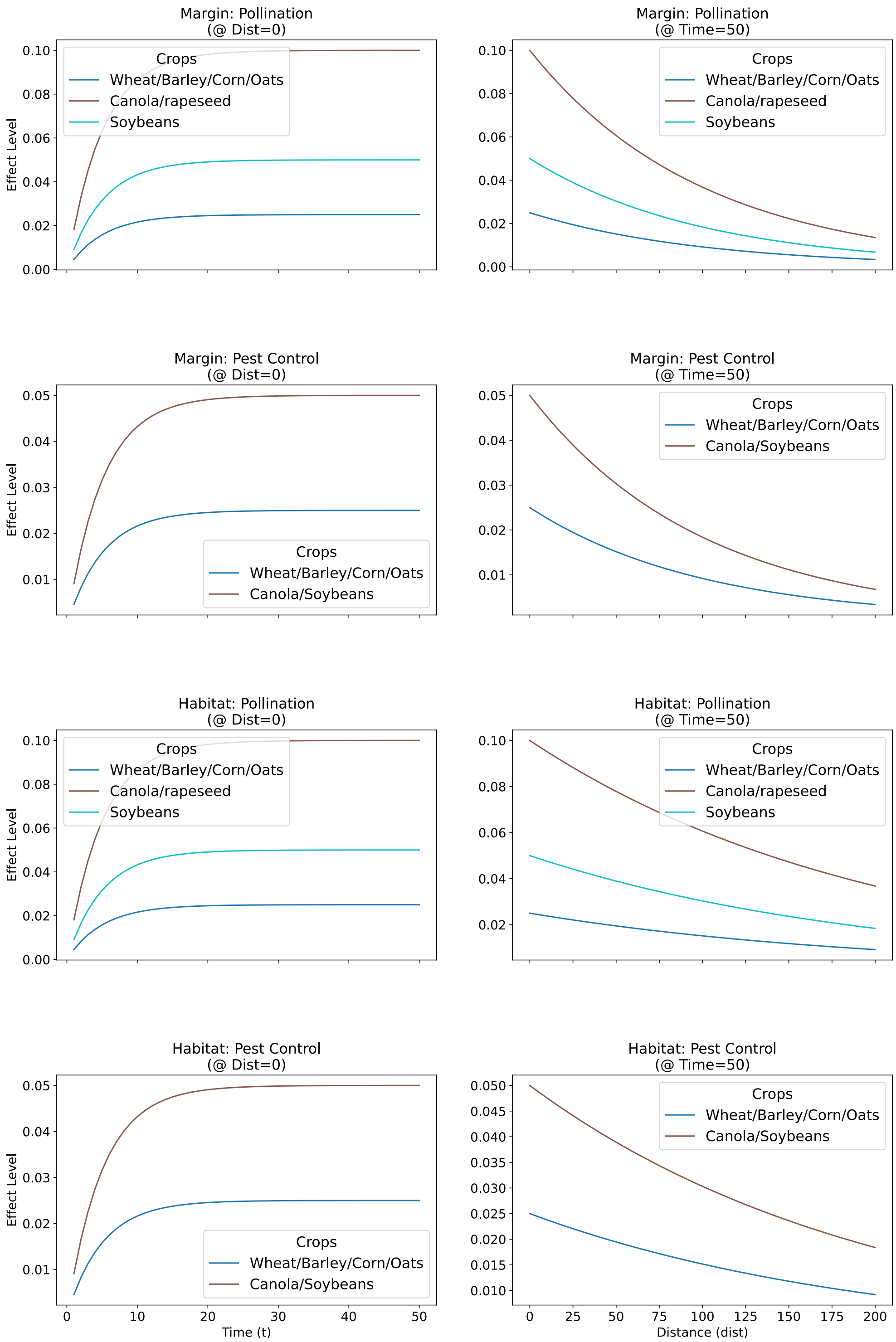

Fig. 7 Effect level as a function of time and distance. Crops are grouped if they share the same parameters. Left panel shows behavior with time, and right panel shows behavior with distance.

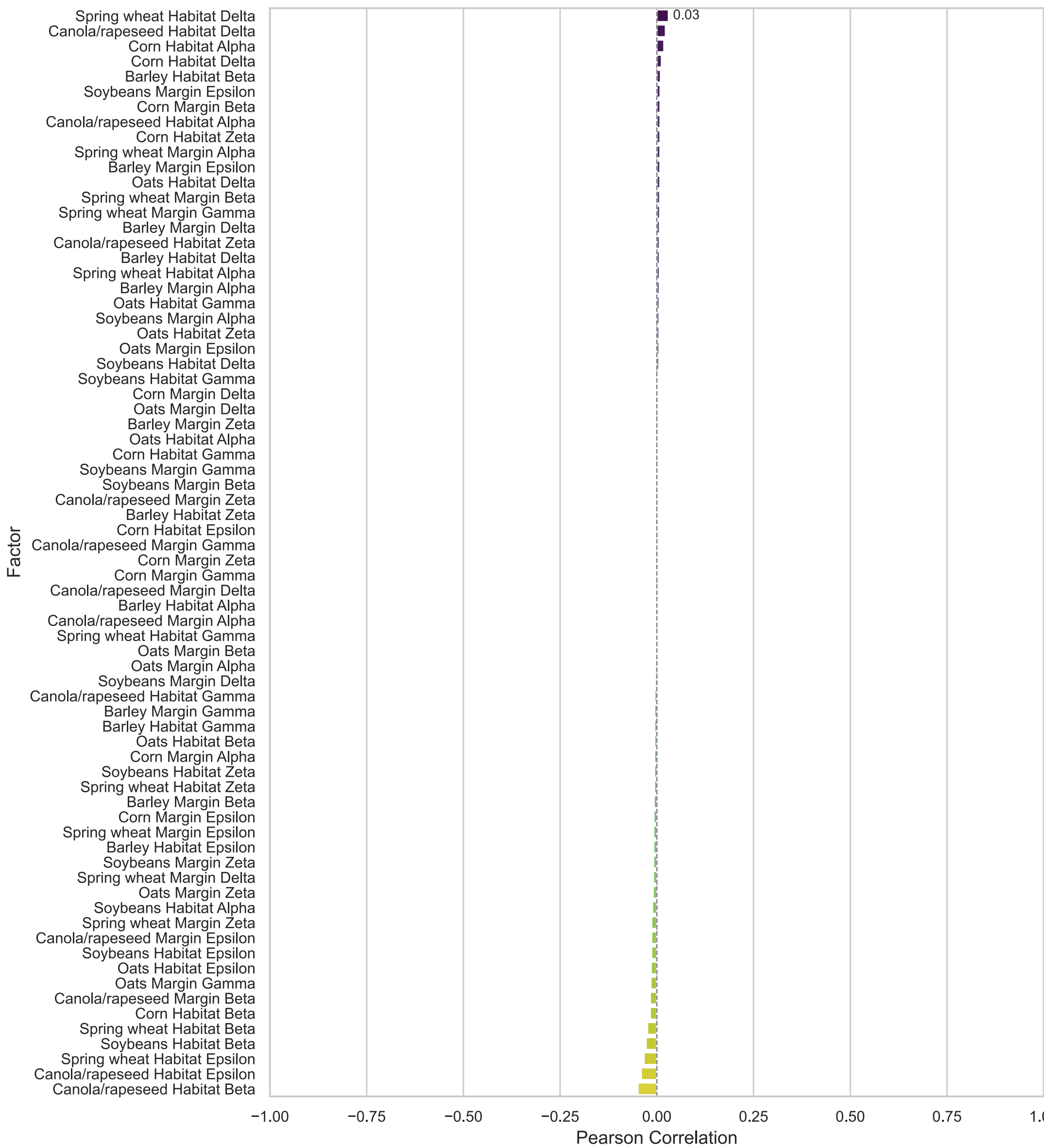

Fig. 8 Sensitivity of habitat interventions to crop specific parameters. Canola/rapeseed and spring wheat parameters show the highest sensitivity, with the rest being negligible.

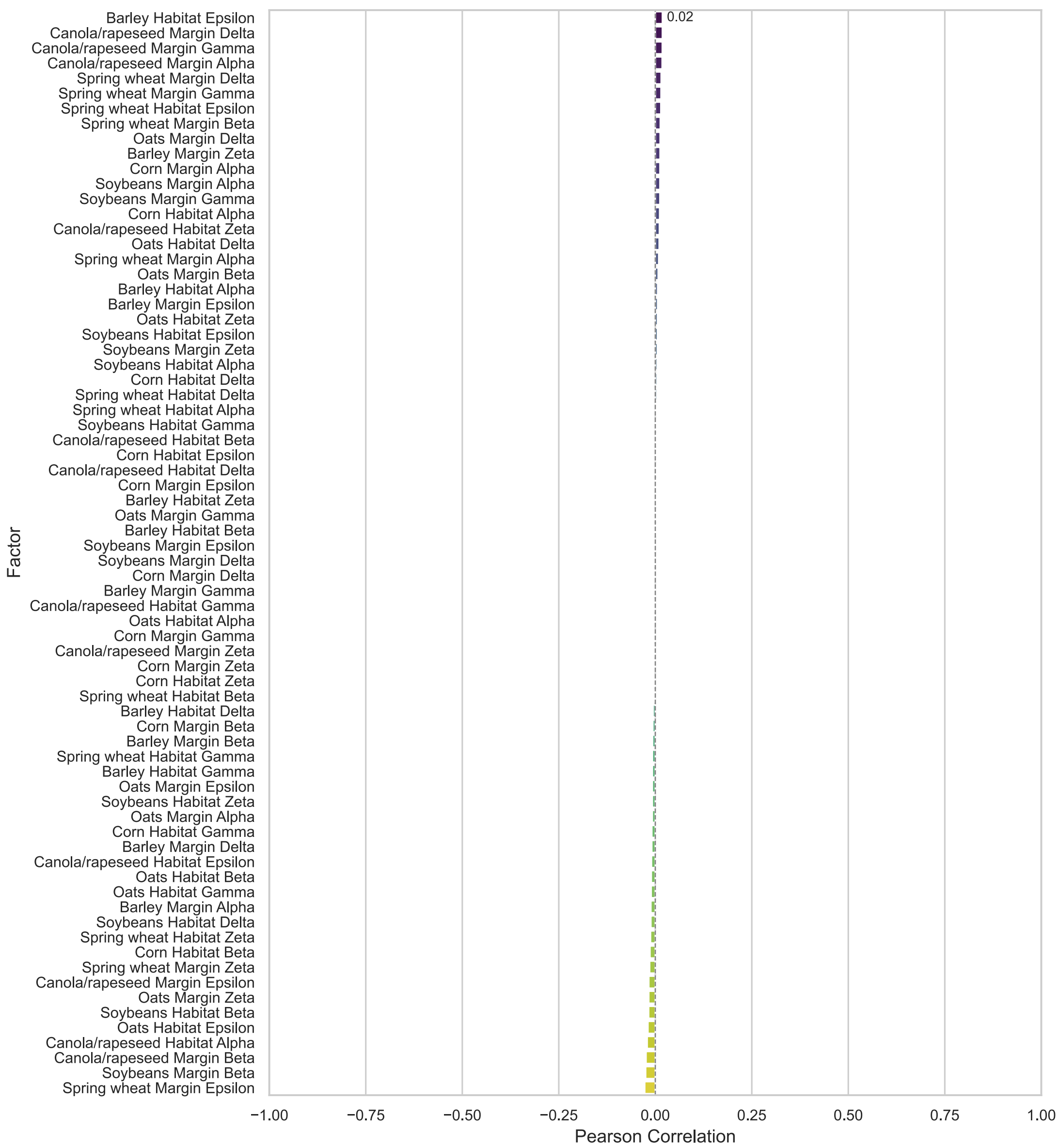

Fig. 9 Sensitivity of margin interventions to crop specific parameters. Most crop parameters have negligible effects on intervention fractions.

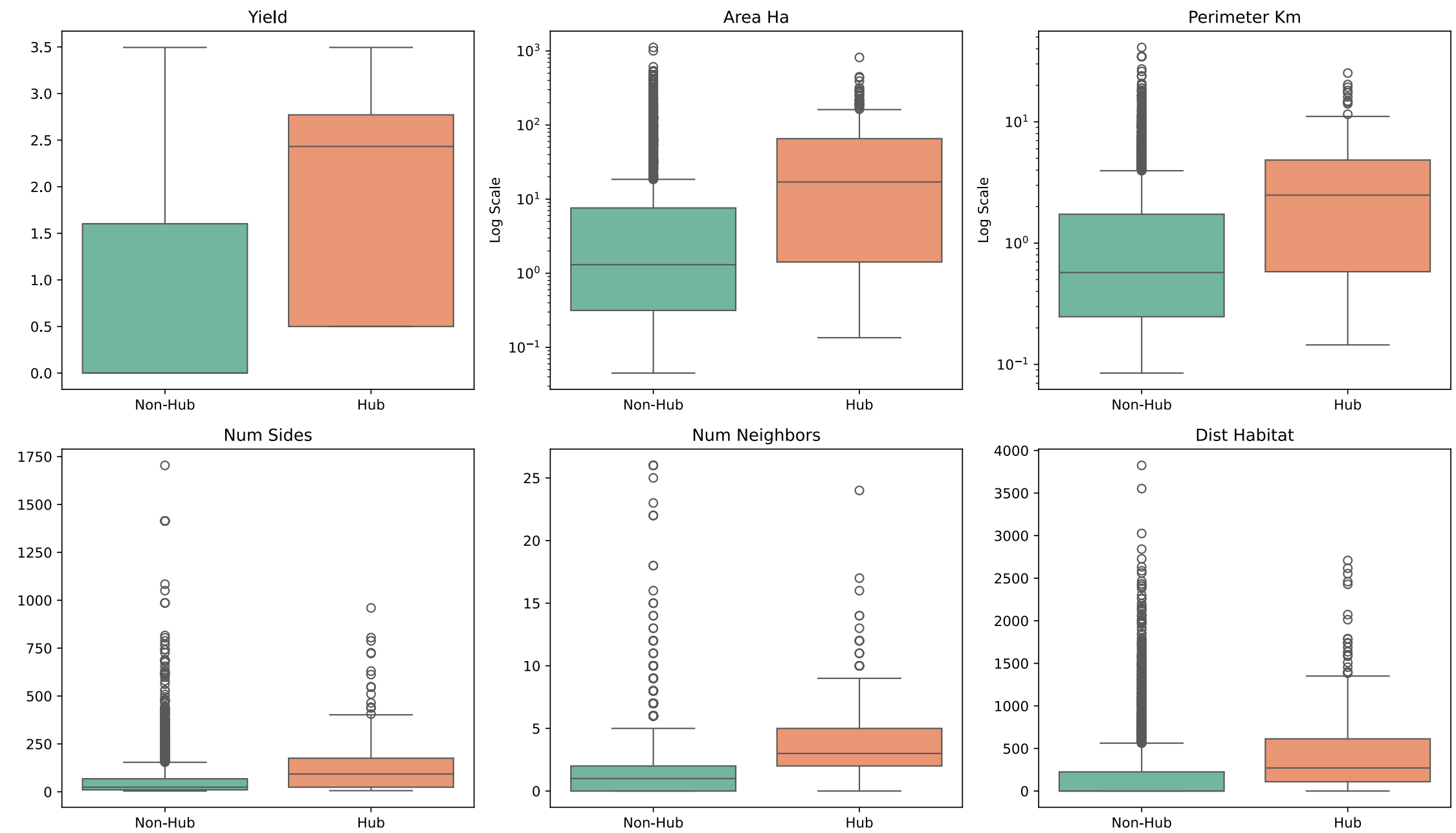

Fig. 10 Box plots of aggregate parameters for hub and non-hub plots. Showing baseline yield, area, perimeter, number of sides, number of neighbours, and distance to habitat.

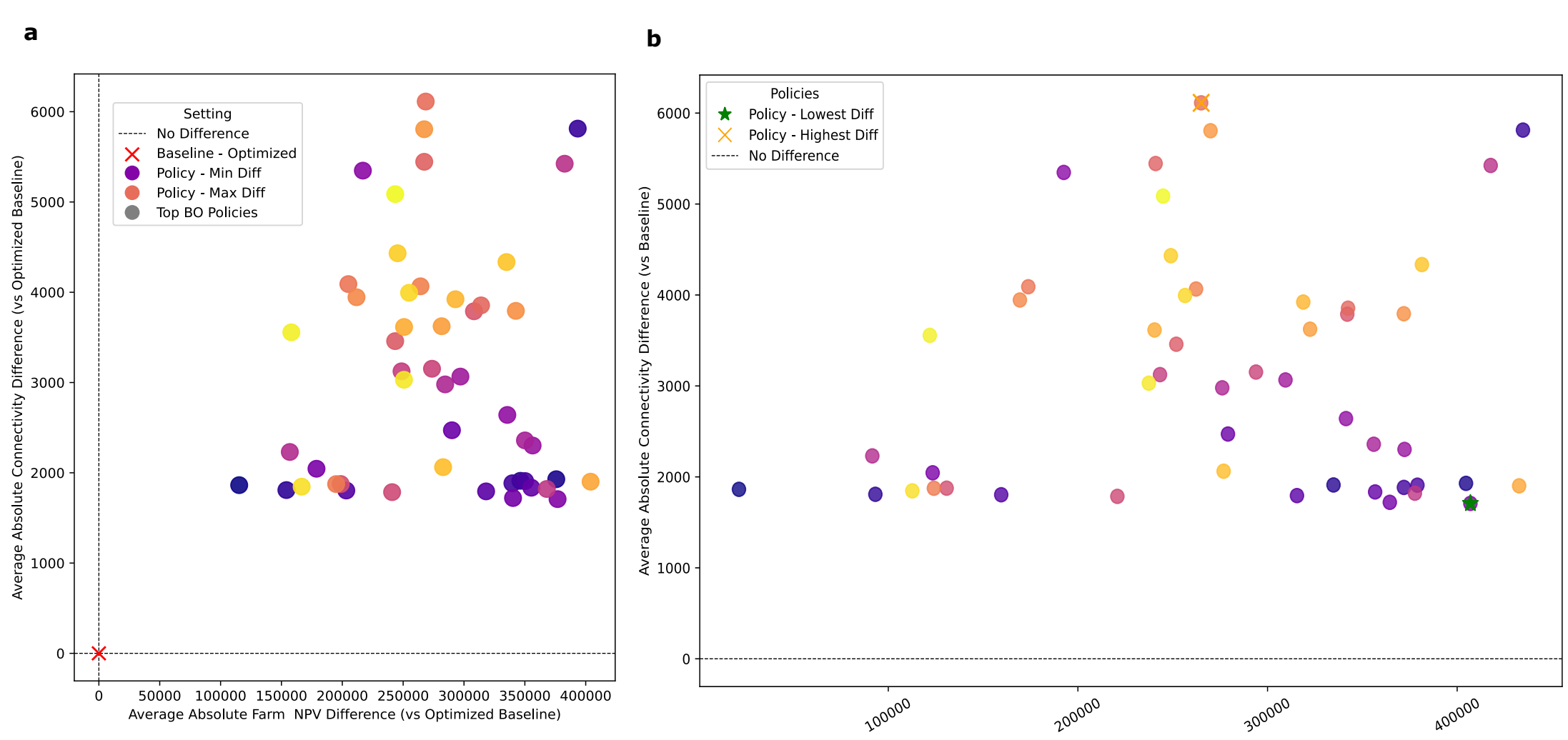

Fig. 11 Scatter plots for top performing policies. a) Average absolute connectivity difference with optimized baseline vs average absolute farm NPV difference with optimized baseline for top 50 policies identified by BO. d) Average absolute connectivity difference with optimized baseline vs average policy cost for top 50 policies identified by BO.

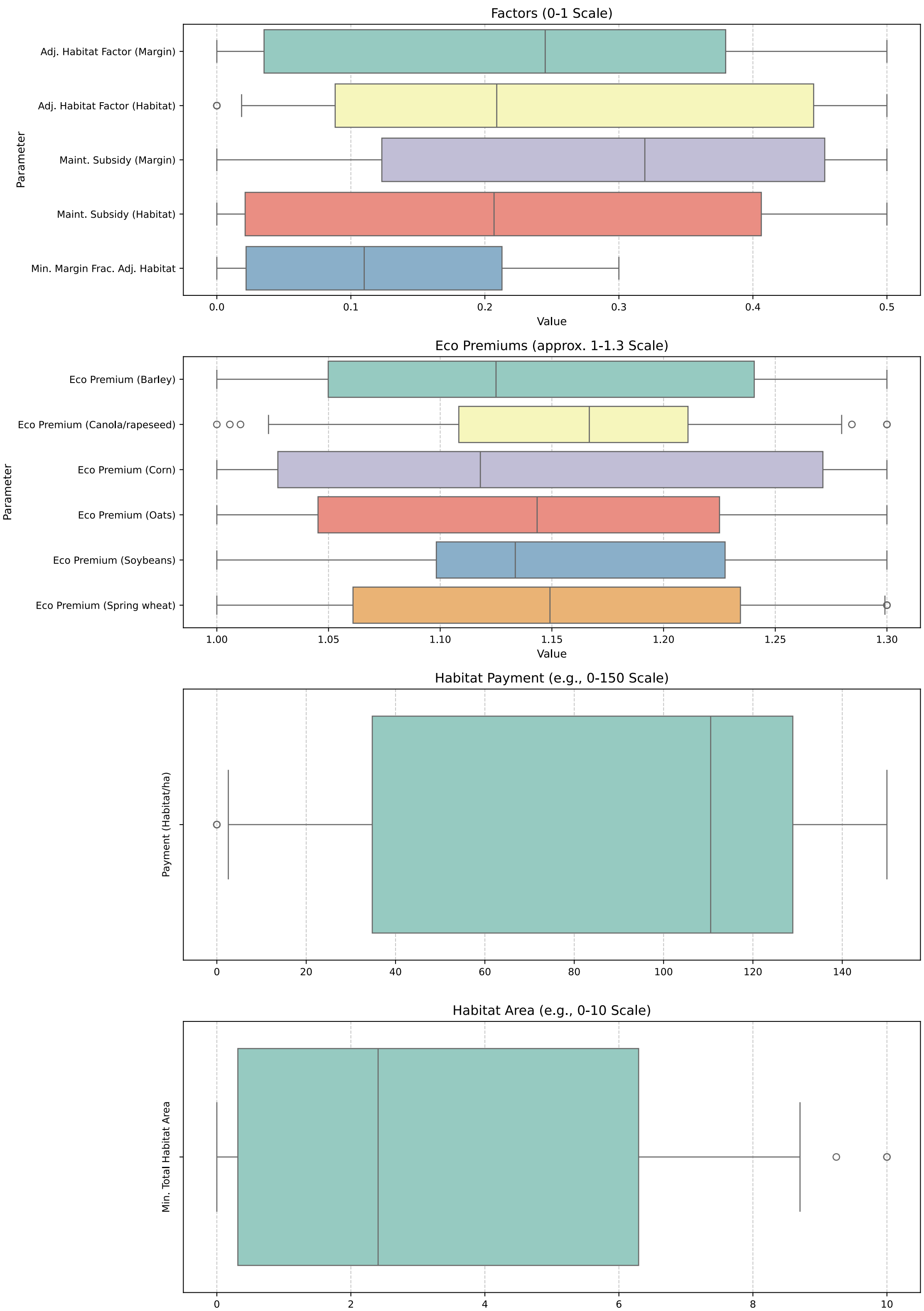

Fig. 12 Key policy parameter distributions in top-performing policies. Policies are grouped according to ranges and include subsidies, payments, mandates, and eco-premiums.

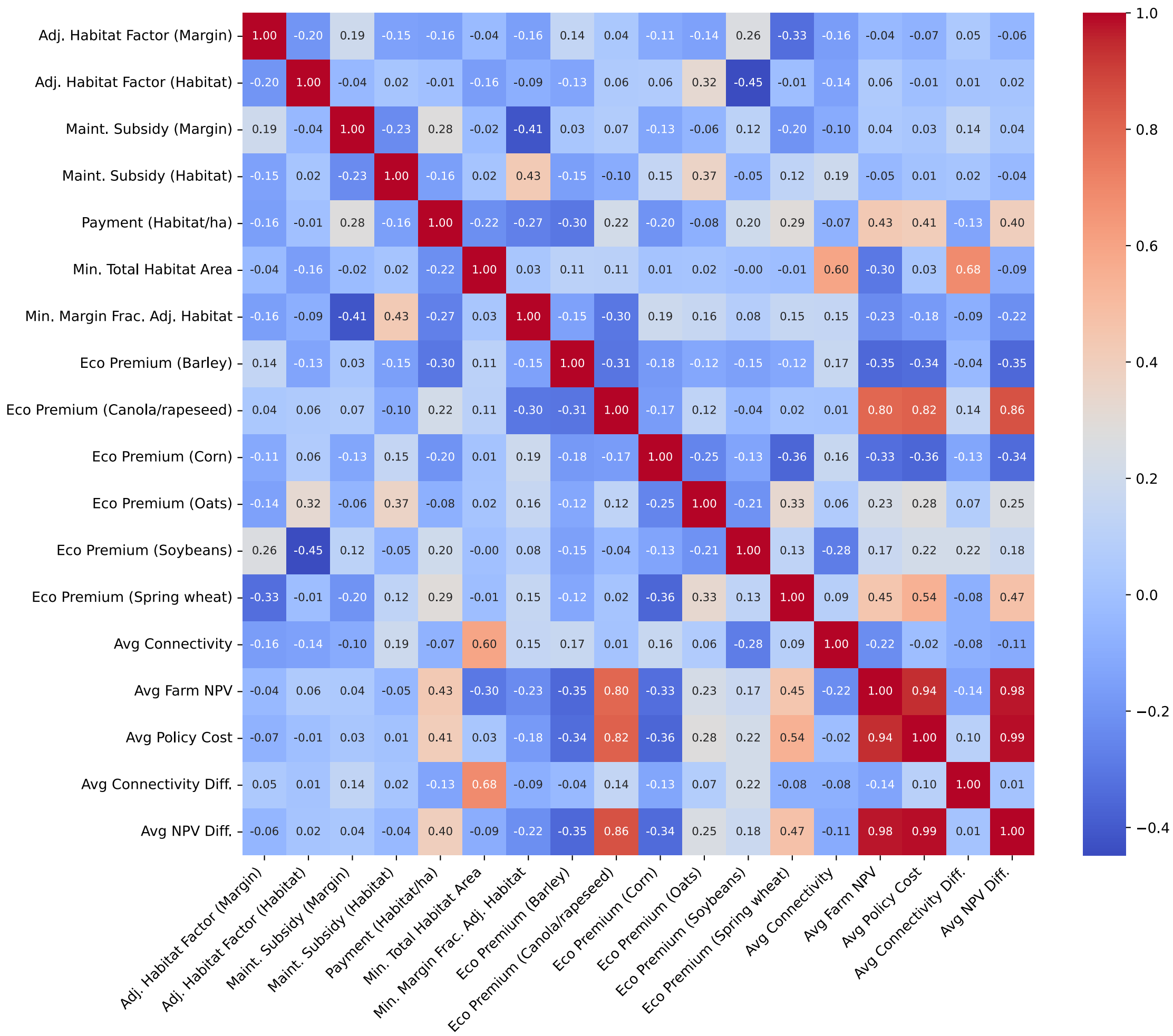

Fig. 13 Correlation matrix of policy parameters and policy metrics. Policy parameters include subsidies, payments, mandates, and eco-premiums, and metrics include connectivity, NPV, policy cost, farm-wise connectivity difference and NPV difference with optimized baseline.

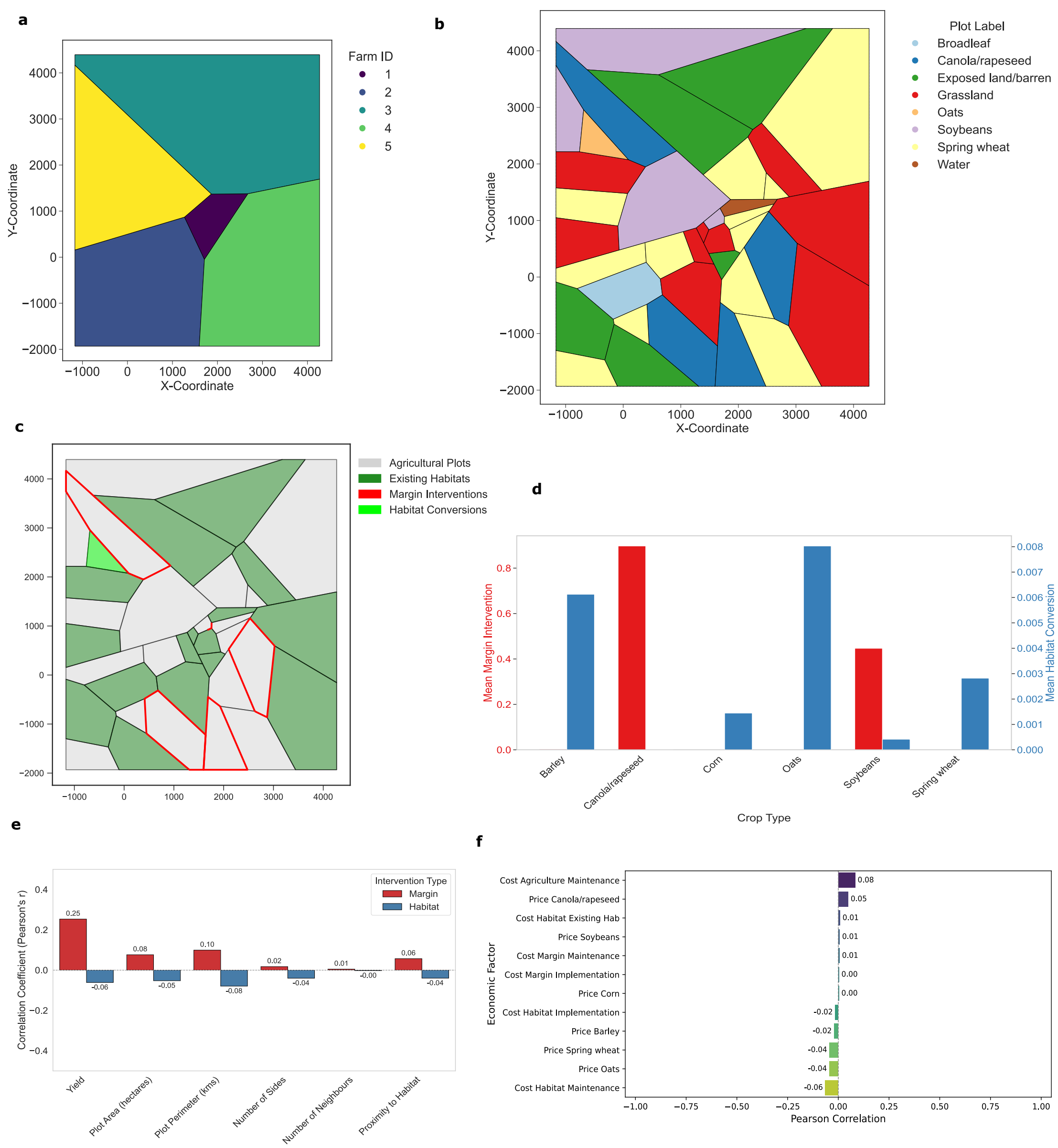

Fig. 14 EI model results on synthetic data. a) Generated synthetic farm-level geospatial data for one of the landscape configurations. b) Generated synthetic plot-level geospatial data. c) Optimal margin and habitat fractions decided by the EI model. d) Margin and habitat intervention fractions chosen based on crop type. e) Pearson correlation coefficients between key plot factors and optimal interventions. f) Sensitivity of habitat conversion levels to crop prices and costs.

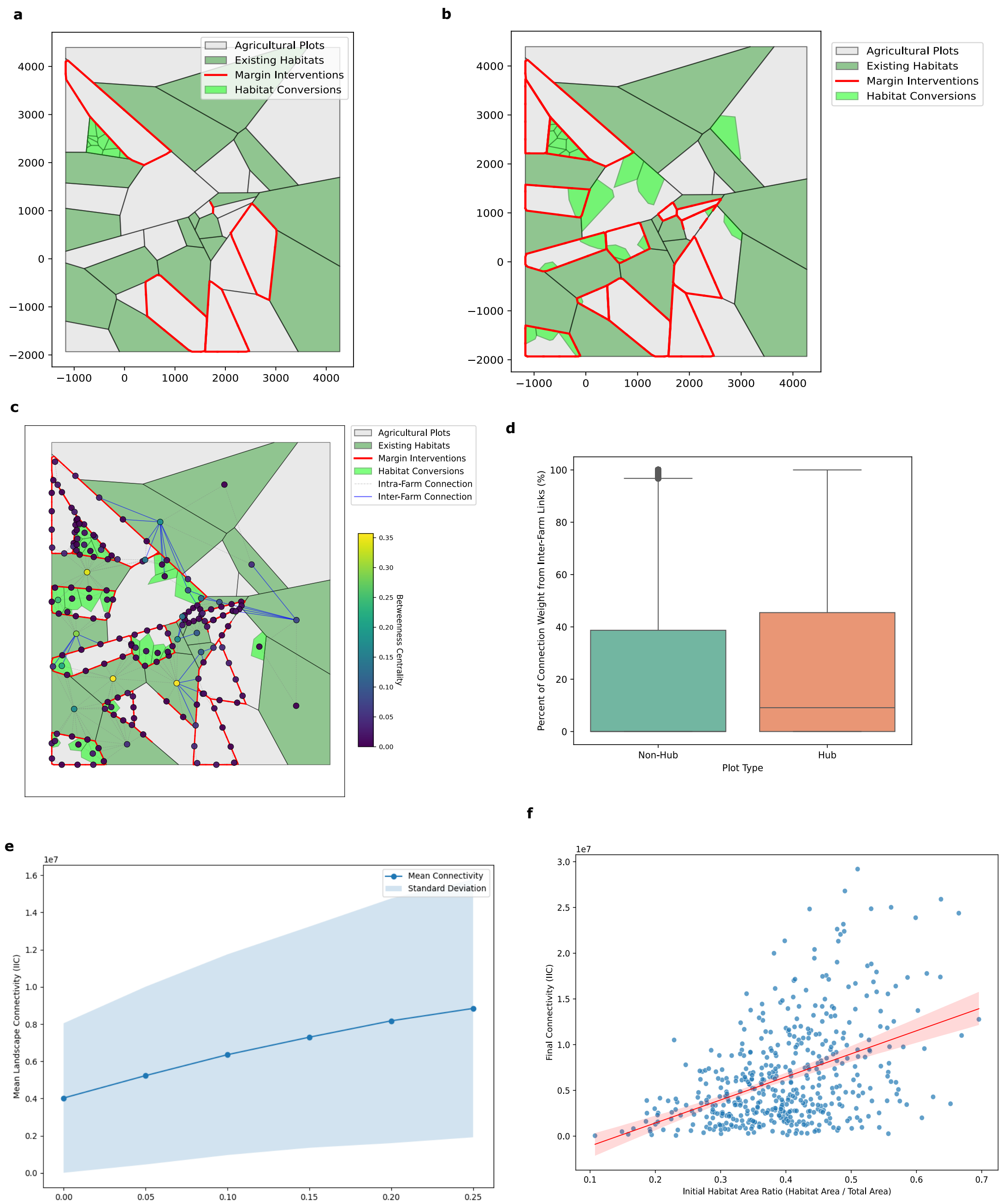

Fig. 15 EC model results on synthetic data. a) Margin arcs and habitats areas chosen after EC repositioning. b) Margin arcs and habitats areas chosen after EC connectivity optimization. c) Plot level betweenness centrality and intra-farm (dashed gray)/inter-farm (solid blue) connections. d) Box plot of aggregate yield for hub and non-hub plots. e) Mean landscape connectivity (IIC) vs maximum allowed NPV loss ratio. f) Scatter plot of optimized connectivity (IIC) vs initial existing habitat area ratio.

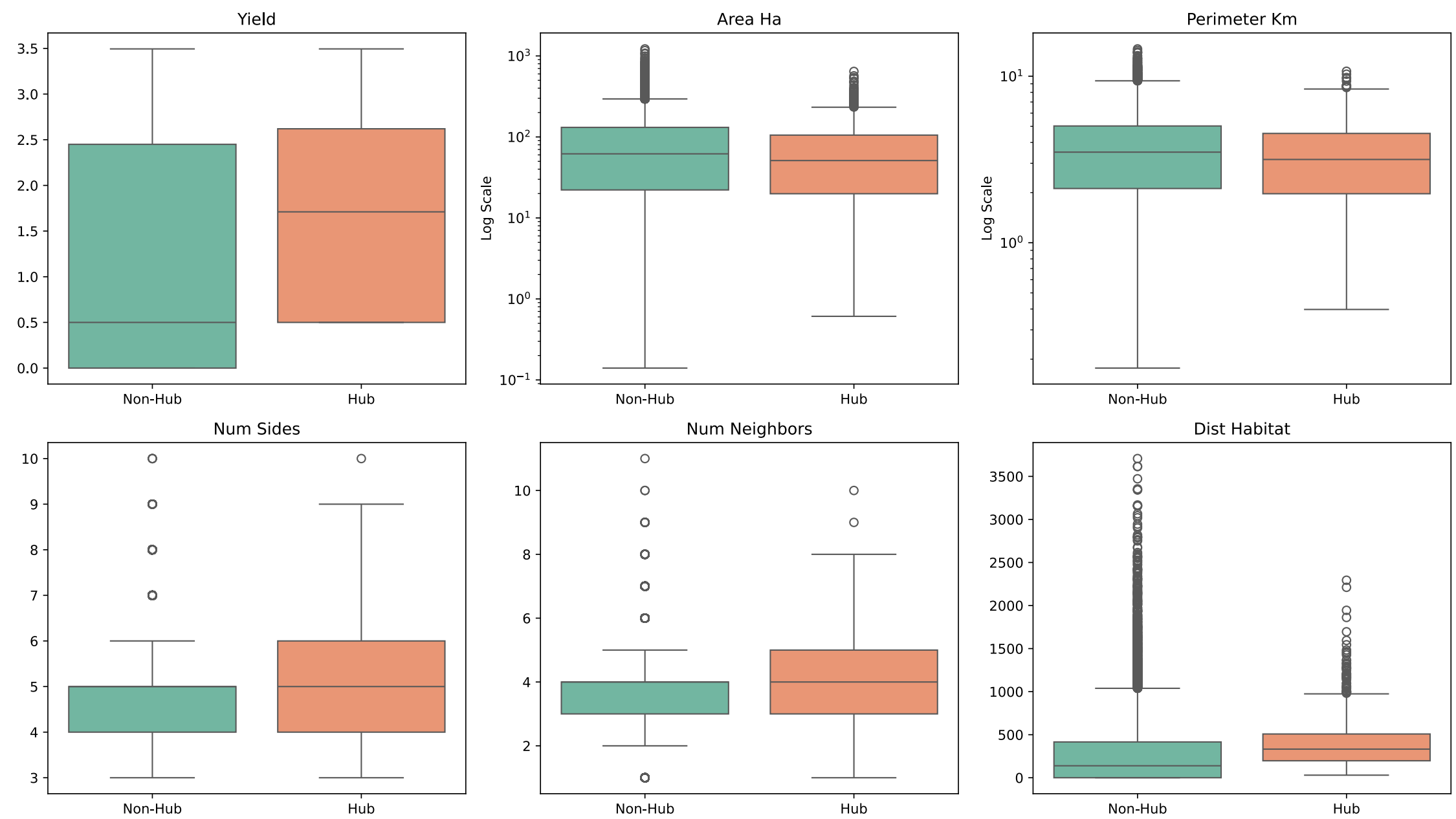

Fig. 16 Box plots of aggregate parameters for hub and non-hub plots on synthetic data. Showing baseline yield, area, perimeter, number of sides, number of neighbours, and distance to habitat.

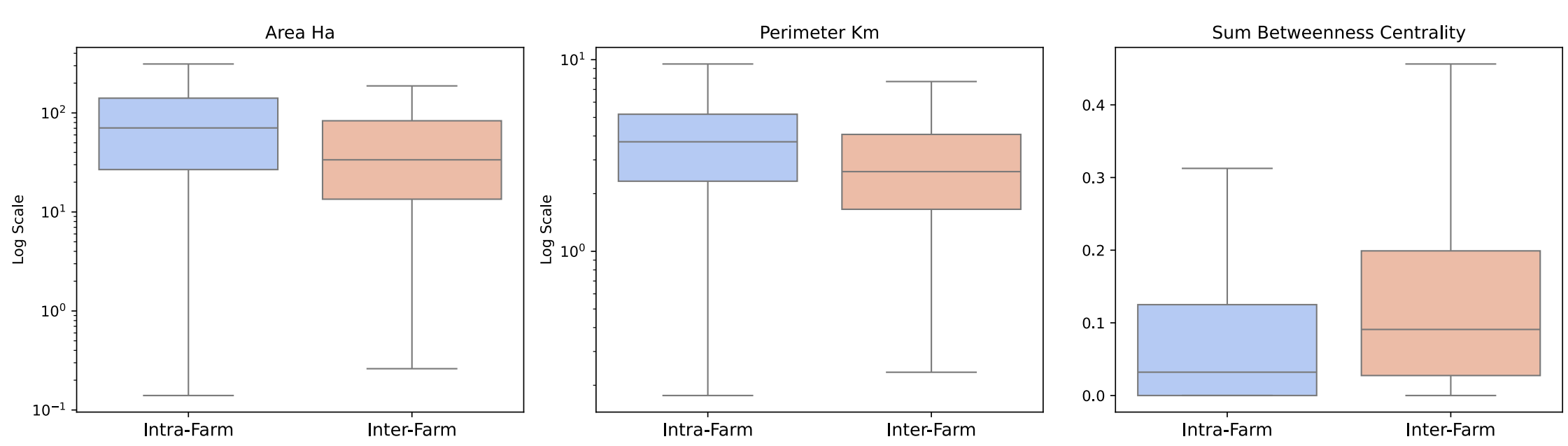

Fig. 17 Box plots of aggregate parameters for plots with intra-farm and inter-farm dominating connections on synthetic data. Showing area, perimeter, and sum betweenness centrality.
